# What is happening at the atomic level during the seed-mediated growth of copper on gold?

**DOI:** 10.1126/sciadv.aee8346

**Published:** 2026-08-05

**Authors:** Yanzi Yan, Kei Kwan Li, Xintong Huang, Xude Yu, Yong Ding, Jianhong Xu, Younan Xia

**Affiliations:** ^1^ School of Chemistry and Biochemistry, Georgia Institute of Technology, Atlanta, Georgia 30332, United States.; ^2^ The Wallace H. Coulter Department of Biomedical Engineering, Georgia Institute of Technology and Emory University, Atlanta, Georgia 30332, United States.; ^3^ The State Key Laboratory of Chemical Engineering and Low-Carbon Technology, Department of Chemical Engineering, Tsinghua University, Beijing 100084, P. R. China.; ^4^ School of Materials Science and Engineering, Georgia Institute of Technology, Atlanta, Georgia 30332, United States.

## Abstract

Bimetallic nanocrystals obtained via seed-mediated growth are typically analyzed using conventional electron microscopy and many interesting features at the atomic level are often missed. Here we report the seed-mediated growth of copper (Cu) on gold (Au) nanospheres for the synthesis of Au-Cu bimetallic nanocrystals enclosed by a Janus surface comprised of an ordered intermetallic phase and a disordered alloy. Initially, the abundant Cu(II) precursor enables uniform Cu deposition, followed by inter-diffusion to generate an ultrathin shell made of intermetallic Au_1_Cu_1_. The growth subsequently evolves into an asymmetric mode, where Cu deposition preferentially proceeds at one activated site to generate Au_x_Cu_y_ alloy with a graded composition. When tested for nitrate reduction, the Janus nanocrystals show 100% Faradaic efficiency across a wide potential range and a high ammonia yield rate.

## Teaser

Seed-mediated growth of Cu on Au nanospheres creates intermetallic-alloy Janus nanocrystals for electrochemical nitrate reduction.

## INTRODUCTION

Bimetallic nanocrystals have garnered significant attention owing to their unique properties often superior to their monometallic counterparts (*1*–*4*). The incorporation of two distinct metals into the same nanocrystal can enrich its localized surface plasmon resonance (LSPR) properties while enhancing its performance in both catalytic and sensing applications (*5*–*7*). Depending on the spatial distributions or arrangements of the elements, one can categorize bimetallic nanocrystals into different groups featuring Janus, core-shell, alloyed, and intermetallic structures, respectively (*8*). Among them, Janus nanocrystals bearing a side-by-side arrangement of two components have been a subject of extensive research owing to their tunable properties for different applications such as self-assembly (*9*), sensing (*10*), and in particular, catalysis (*11*–*13*). As a notable example, catalysts based on Janus nanocrystals allow a complex reaction to occur on the regions with different compositions, in parallel or sequentially, and then combine to give the highest efficiency (*14*, *15*).

Owing to its low price and the appropriate adsorption strength of nitrate (NO_3_^−^), Cu has emerged as one of the most promising catalytic materials toward electrochemical nitrate reduction reaction (NO_3_RR) (*16*, *17*). As an important renewable energy carrier, ammonia (NH_3_) can contribute to reducing carbon emissions and mitigating global warming when produced through sustainable pathways (*18*), and NO_3_RR offers a promising alternative to the conventional carbon dioxide-intensive Haber-Bosch process by converting nitrate pollutants into NH_3_ under mild reaction conditions. However, the limited capability of Cu to dissociate water tends to result in poor selectivity toward NH_3_ production. To overcome this limitation, integrating Cu with another metal capable of water dissociation in the configuration of a Janus structure offers an effective strategy to improve the selectivity. Specifically, M-Cu (M = Ni, Ru, and Co) Janus nanocrystals have been reported to exhibit a Faradaic efficiency (FE) over 90% toward NH_3_ during NO_3_RR (*19*–*21*). However, these electrocatalysts involving transition metals are plagued by poor stability, and one potential solution to this issue is to replace the transition metal with a noble metal such as Au.

Seed-mediated growth is a versatile route to the synthesis of Au-Cu Janus nanocrystals (*22*, *23*). Specifically, asymmetric Cu deposition on preformed Au seeds has been associated with factors such as controlling the reduction kinetics, leveraging the large lattice mismatch between Au and Cu, and/or selecting seeds with defects, for the fabrication of Au-Cu Janus nanocrystals (*24*). For example, Jia *et al.* (*25*) demonstrated that the large (11.4%) lattice mismatch between Au and Cu, accompanied by the slow reduction of Cu(II) precursor at a low concentration, led to the asymmetric deposition of Cu on Au bipyramids for the synthesis of Au-Cu Janus nanocrystals. However, all the Au-Cu Janus nanocrystals reported in the literature were only characterized using conventional electron microscopy, including elemental mapping by energy-dispersive x-ray spectroscopy (EDS) to give a side-by-side arrangement for the Au and Cu components. Many interesting features related to the atomic details were inevitably missed or overlooked.

**Fig. 1.**
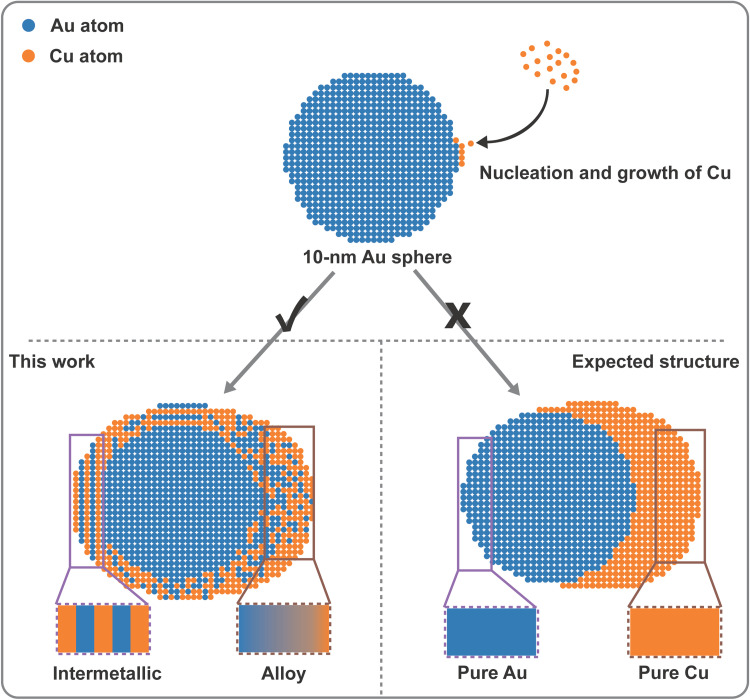
Structure and characteristics of the Janus nanocrystals. Schematic illustration of the Janus nanocrystal with an intermetallic phase on one side and an alloy phase on the other. This is completely different from the expected Janus structure with Cu and Au regions being exposed side by side on the surface, as reported in the literature based upon conventional electron microscopy analysis.

Herein, we report an atomic understanding of the synthesis of Au-Cu bimetallic nanocrystals with an unusual Janus surface by leveraging the inter-diffusion between the deposited Cu atoms and the underlying Au atoms (*26*). As illustrated in Fig. 1, our results suggest a picture completely different from what has been reported in the literature. Our systematic characterizations reveal that a two-stage growth mechanism is involved in the formation of such a Janus surface. In the initial stage, the Cu atoms are evenly deposited on the entire surface of each Au nanosphere to form an ultrathin shell made of the Au_1_Cu_1_ intermetallic. As the reaction proceeds, the deposition of Cu atoms is switched to an asymmetric mode. One side of the intermetallic shell with greater accumulation of Cu is transformed into an alloy, while the other side remains in the intermetallic phase. Notably, such Janus nanocrystals show remarkable catalytic performance toward NO_3_RR.

## RESULTS

### Synthesis and characterizations

We first prepared spherical Au seeds with an average diameter of 10.8 ± 0.3 nm by following a reported protocol (fig. S1) (*27*). The nanospheres were directly utilized as seeds for the synthesis of Janus nanocrystals (see Materials and Methods, and table S1) (*28*, *29*). By adjusting the number of Au seeds added into the reaction mixture, the amount of Cu deposited on each Au sphere could be readily tuned. Specifically, when 3.11 × 10^10^ of Au nanospheres were added (the standard synthesis), the resulting Au-Cu bimetallic nanocrystals had the largest volume of Cu. Increasing the number of Au nanospheres by 2-, 3-, and 4-fold reduced the amount of the deposited Cu accordingly. In our discussion, the products are denoted x1, x2, x3, and x4 samples, respectively.

**Fig. 2.**
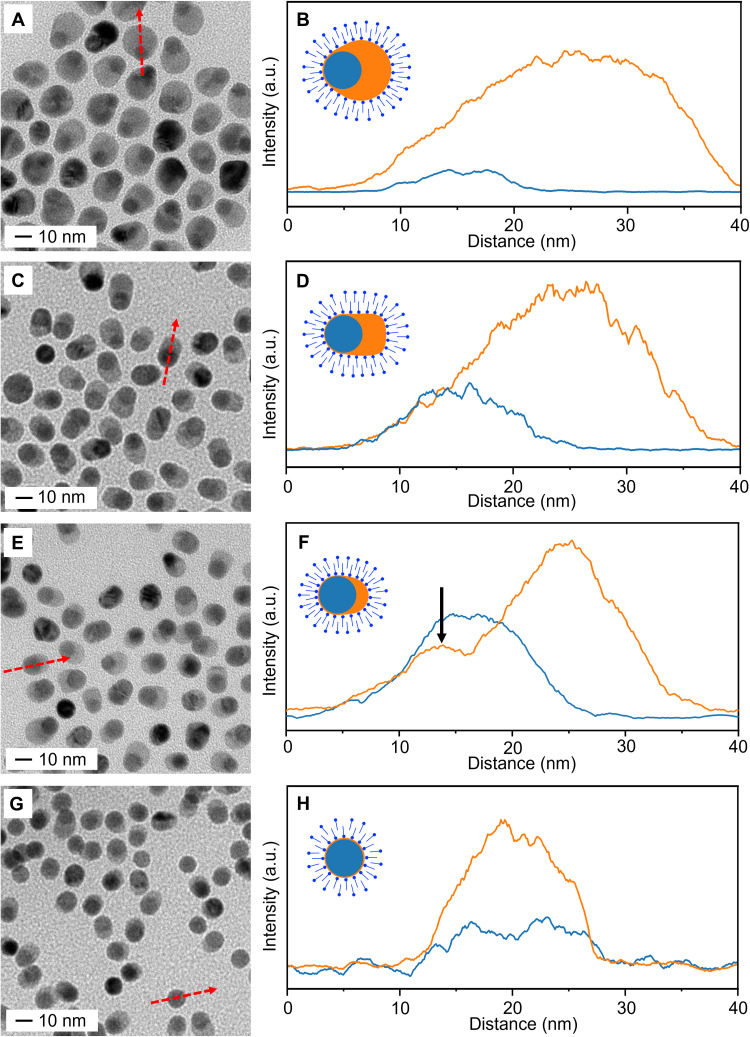
Synthesis and characterizations of the Au-Cu bimetallic nanocrystals. TEM images and EDS line-scanning profiles of the Au-Cu bimetallic nanocrystals synthesized by introducing different numbers of the 10-nm Au seeds relative to the standard synthesis (3.11 × 10^10^): (**A** and **B**) x1, (**C** and **D**) x2, (**E** and **F**) x3, and (**G** and **H**) x4, respectively. The blue and orange curves correspond to Au and Cu, respectively. The red arrows indicate the direction of EDS line-scanning across the particle over a distance of 40 nm. The insets show schematics of the corresponding nanocrystals.

We initially characterized the samples using transmission electron microscopy (TEM). As shown in Fig. 2A for the x1 sample, Cu was unevenly deposited on the surface of each Au seed, with one side of the particle being covered by more Cu than the other. This uneven distribution of Cu was further confirmed by EDS mapping and line-scanning in fig. S2A and Fig. 2B, respectively (*30*). When comparing the Au and Cu line-scanning profiles, their signals overlapped across the particle, suggesting that Cu was deposited on the entire surface of the Au nanosphere, but in an asymmetric distribution (see the inset). Taken together, we argue that Cu asymmetrically nucleated and grew from the surface of each seed for the generation of a “Janus” nanocrystal. Such “Janus” nanocrystals have also been reported in the literature for the growth of Cu on Au or Pd seeds (*31*, *32*). As detailed in the following sections, however, these reported structures are completely different from ours.

When the number of Au seeds was increased by 2-fold, the amount of Cu deposited on each particle was reduced proportionally (Fig. 2C and fig. S2B). The EDS line-scanning profile of an individual particle again confirmed that the Cu was deposited on the entire Au surface (Fig. 2D). Increasing the number of Au seeds by 3-fold, the amount of deposited Cu was further reduced (Fig. 2, E and F, and fig. S2C). Interestingly, a small bump in Cu signal was observed in the EDS line-scanning profile (fig. S3A). This observation implied that an ultra-thin shell containing Cu was formed on the Au seed, motivating us to further analyze the x3 sample. A more detailed discussion on their atomic structure and growth mechanism can be found in the following section. As for the x4 sample, two distinct types of particles were observed: (i) Au seeds with very little Cu and (ii) Au seeds with essentially no Cu (Fig. 2G and fig. S2D). The line-scanning profile of the second type of particle suggested the formation of an ultrathin shell containing Cu on the Au seed (Fig. 2H) despite its absence under TEM imaging. The discrepancy between the TEM and EDS line-scanning analyses can be attributed to the limited capability of conventional TEM in resolving a shell as thin as a few atomic layers only.

**Fig. 3.**
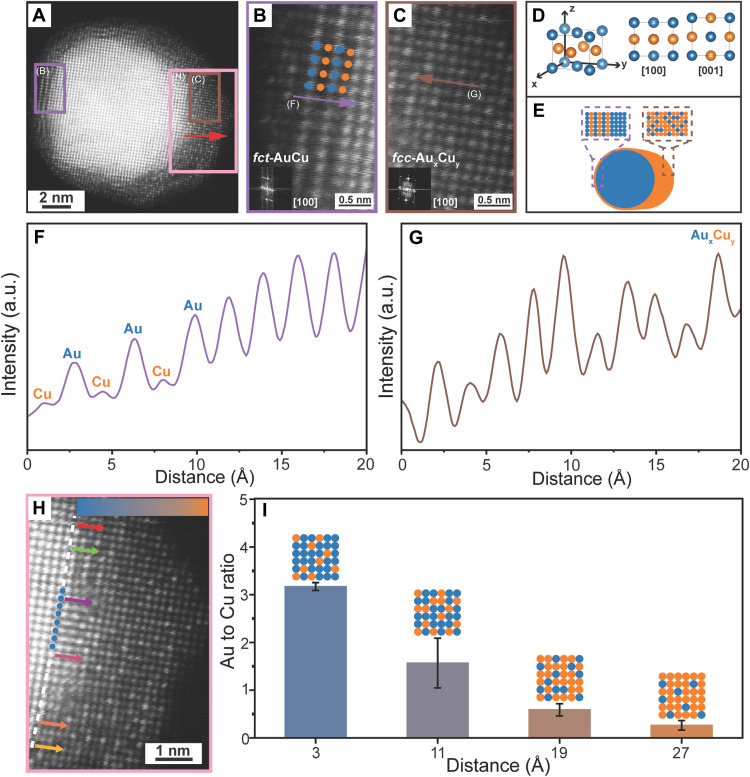
Characterizations of the x3 sample. (**A**) HAADF-STEM image along the [100] zone axis; (**B**) magnified HAADF-STEM image, with the inset showing a fast Fourier transform (FFT) pattern of the left portion of the shell; (**C**) magnified HAADF-STEM image, with the inset showing an FFT pattern of the right portion of the shell; (**D**) atomic model of an *fct*-AuCu lattice, together with the atomic pattern viewed along [100] and [001] directions; (**E**) schematic illustration of the atomic structures of a particle from the x3 sample; (**F**) atomic intensity profile along the purple arrow in (B), showing an ordered arrangement of the Au and Cu atoms in the left portion of the shell; (**G**) atomic intensity profile along the brown arrow in (C), showing a random arrangement of the Au and Cu atoms in right portion of the shell; (**H**) magnified HAADF-STEM image used to obtain the plot in (I); with the arrows indicating the paths to acquire atomic intensity profiles; and (**I**) Au to Cu atomic ratio in the *fcc*-Au_x_Cu_y_ alloy shell as a function of distance from the dot line in (H). Orange: Cu; Blue: Au.

To resolve the atomic structure of the bimetallic nanocrystals in the x3 sample (fig. S3B), we switched to high-angle annular dark-field scanning transmission electron microscopy (HAADF-STEM). As shown in Fig. 3A, the HAADF-STEM image of a particle viewed along the [100] direction indicates a clear core-shell structure. Although the core exclusively showed bright atoms corresponding to Au, the shell contained atoms with different contrasts, suggesting the presence of both Au and Cu. The formation of a bimetallic shell can be attributed to the inter-diffusion between the deposited Cu and the underlying Au, a scenario well-documented in the literature (*33*, *34*). Interestingly, the atoms in different regions of the shell exhibited distinct orderings. An intermetallic structure was observed in the region with less Cu deposition (Fig. 3B), whereas an alloy was formed in the region with more Cu deposition (Fig. 3C). In particular, the alternating Au-Cu atomic pattern in Fig. 3B matched the theoretical model of face-centered-tetragonal (*fct*)*-*AuCu along [100], which was the zone axis for STEM imaging (Fig. 3D). It should be noted that the axes in this *fct* lattice cell were defined differently from those in a primitive cell in order to align with the zone axis (fig. S4). Conversely, the pattern shown in Fig. 3C did not align with any models of Au-Cu intermetallic phases, including *fct*-AuCu, face-centered-cubic (*fcc*)-Au_3_Cu, and *fcc*-AuCu_3_ along the [100] direction. Taken together, we conclude that the Au-Cu bimetallic nanocrystals had an Au core and a Janus shell, with one region of the shell made of *fct*-AuCu intermetallic phase and the rest comprised of *fcc*-Au_x_Cu_y_ alloy (Fig. 3E). We further acquired the atomic intensity profiles along the purple and brown arrows in Fig. 3, B and C, to confirm this unique Janus core-shell structure. The atomic intensity profiles were processed using Origin software with a consistent 10-point smoothing procedure, and Au and Cu atomic columns were assigned based on the intensity in the profiles. As shown in Fig. 3F, the brighter Au atoms and dimmer Cu atoms exhibited an ordered structure characteristic of *fct*-AuCu phase. In contrast, the disordered pattern in Fig. 3G implied the formation of an alloy. In the alloy region, each projected atomic column contained both Au and Cu due to atomic inter-diffusion, and therefore the HAADF-STEM intensity reflected an averaged atomic contrast rather than distinct elemental columns.

Interestingly, we observed a gradation in terms of Au to Cu atomic ratio in the alloy region (*35*). Specifically, we examined six atomic intensity profiles along the arrows in Fig. 3H, and the results are summarized in fig. S5. This statistical analysis of the Au to Cu ratio reveals a gradual decrease in the relative Au content and an increase in Cu content when moving from the inner to the outer surface of the shell (Fig. 3I). It should be noted that due to the projection effect in a HAADF-STEM image, the Vegard’s law analysis should be considered a qualitative compositional trend rather than precise stoichiometry.

### Mechanistic understanding

The formation of an asymmetric shell uneven in thickness can be attributed to the kinetics involved in the Cu(II) reduction and strain-induced Stranski-Krastanov (S-K) growth mode (*36*–*38*). Specifically, the concentration of the Cu(II), that is, [Cu(II)] at any time point *t* can be expressed by the plot and equation in fig. S6A. According to the plot, the reduction rate of Cu(II) peaked at the onset (*t_0_*) of the synthesis, leading to rapid production of Cu atoms within a short period of time. The adequate supply of atoms in the initial stage allowed all the reactive sites on the Au seed to receive Cu atoms at roughly the same rate. In addition, the easy wetting of Cu on Au favored a complete coverage of Cu over the formation of Cu islands on the Au seeds during the early stage (*39*). As a result, the Cu atoms were uniformly and symmetrically deposited on the surface of an Au seed to generate an ultrathin shell uniform in thickness. Our recent study also suggested that the high-index facets on the Au nanospheres and their relatively small areas were instrumental to the formation of such a uniform, ultrathin shell (*40*). The high propensity of inter-diffusion between Au and Cu atoms caused some regions of the shell to evolve from Cu to the *fct*-AuCu intermetallic phase (*26*, *41*, *42*). As a result, particles with an ultrathin shell comprised of *fct*-AuCu were formed before reaching *t_asym_*. When the synthesis passed *t_asym_*, the reduction rate of Cu(II) would drop below a threshold, causing an inadequate supply of Cu atoms to all the growing sites on the nanocrystal. As a result, the deposition of Cu atoms would take place at certain sites on the surface of the particle only, leading to the formation of under-coordinated Cu atoms with high surface energies (*24*). As such, these sites became activated, favoring further deposition of Cu atoms to break the symmetry of the shell.

In parallel, according to the S-K model, a pseudomorphic two-dimensional (2D) layer growth mode was also favored for the Cu atoms in the early stage (*38*). As the strain gradually accumulated during the thickening of the 2D layer in *fct*-AuCu intermetallic structure, the growth of the shell would be switched from a pseudomorphic 2D layer growth mode to a 3D island mode. Since the particle had a small surface area, only one island would be likely formed on each particle rather than multiple islands (fig. S6B). Once the island was formed, it could serve as the “activated site” for subsequent deposition of Cu, leading to the evolution of an asymmetric shell. Notably, these two mechanisms might both contributed to the observed asymmetric growth behavior. Once an “activated site” was established on the surface of nanocrystals, it was the competition between atom deposition and surface diffusion that ultimately determined the asymmetrical growth of the shell (*43*). In this study, surface diffusion of adatoms remained limited due to the relatively low temperature (100°C) used in our synthesis (*44*). When the deposition rate of Cu atoms was faster than the surface diffusion rate of Cu adatoms, the as-deposited Cu atoms would accumulate at the activated site rather than migrate to other regions on the particle surface, promoting the formation of the asymmetric shell (*43*). Consequently, the Au atoms at the activated sites kept alloying with the newly-deposited Cu atoms, changing the *fct*-AuCu intermetallic phase to an *fcc*-Au_x_Cu_y_ alloy because continuous inter-diffusion could no longer accommodate the stoichiometric ratio of the *fct*-AuCu intermetallic structure. Since the shell at early stage contained both Au and Cu atoms, the Cu signal in the EDS line-scanning profile displayed a small bump with a distance similar to that of the Au signal (i.e., Stage I in fig. S6C). The asymmetric deposition of Cu, together with Au-Cu alloying, generated a larger bump in the EDS line-scanning profile (i.e., Stage II in fig. S6C). Altogether, the distinct shell structures formed at different stages of a synthesis resulted in the EDS line-scanning profile shown by the solid orange trace in fig. S6C, consistent with what was recorded for a particle from the x3 sample (Fig. 2F).

**Fig. 4.**
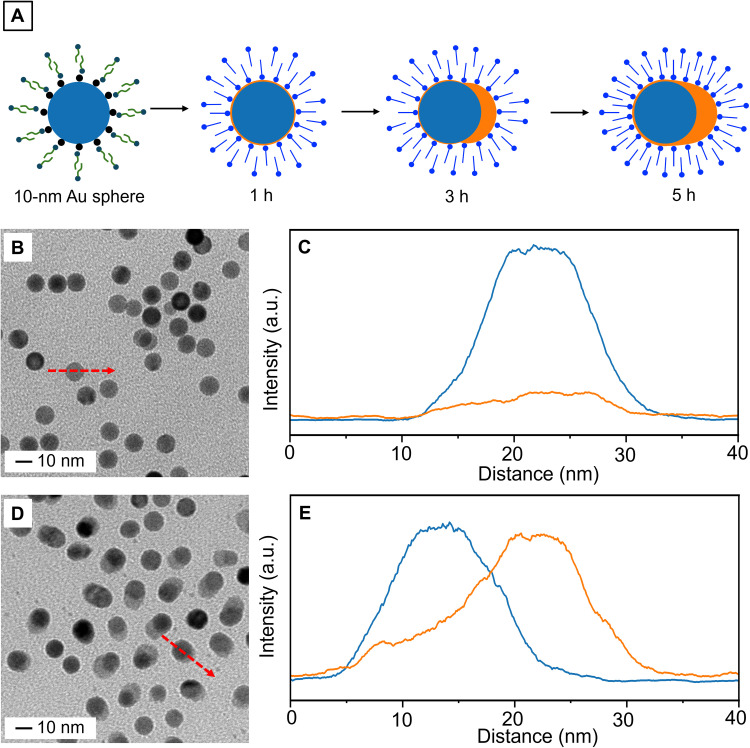
Growth process of the x3 sample. (**A**) Schematic illustration showing the deposition of Cu on a 10-nm Au sphere during the initial 5 hours of a synthesis of the x3 sample. (**B** to **E**) TEM images and EDS line-scanning profiles of the [(B) and (C)] x3 (1 hour) and [(D) and (E)] x3 (3 hours) particles obtained at 1 and 3 hours into the synthesis. Blue lines: Au and orange lines: Cu.

To better understand the formation of the Janus shell, we also analyzed the Au-Cu bimetallic nanocrystals obtained at different stages of a synthesis of the x3 sample (Fig. 4A). At *t* = 1 hour, the particles appeared to be identical to the original Au seeds (Fig. 4B), with no shell on the particles. However, the EDS line-scanning profile in Fig. 4C showed a uniform and steady Cu signal (orange trace) across the particle. This experimental data was in agreement with the theoretical profile illustrated in fig. S6C (Stage I), indicating the formation of a complete shell containing both Au and Cu atoms at this early stage. As the synthesis was prolonged to 3 hours, the particles showed an asymmetric shell due to the uneven deposition of Cu (Fig. 4D). The corresponding EDS line-scanning profile in Fig. 4E confirmed that one side of the particle had more Cu than the other side. Nonetheless, a small bump with a distance similar to that of the Au core was observed in the line-scanning profile, in agreement with the theoretical profile depicted in fig. S6C (Stage II). These samples are hereafter denoted x3 (1 hour) and x3 (3 hour), corresponding to the syntheses terminated at *t* = 1 and 3 hours, respectively. Unless otherwise specified, the x3 sample refers to the synthesis terminated at *t* = 5 hours. Overall, the EDS line-scanning profiles of the Au-Cu bimetallic nanocrystals obtained at different stages of a synthesis, together with their TEM images, support the mechanistic details outlined in fig. S6. In addition, this mechanism was supported by the ultraviolet-visible (UV-vis) spectroscopy data in figs. S7 and S8, with Au nanospheres and Au@Cu core-shell nanocrystals (fig. S9) serving as references (*45*–*48*).

**Fig. 5.**
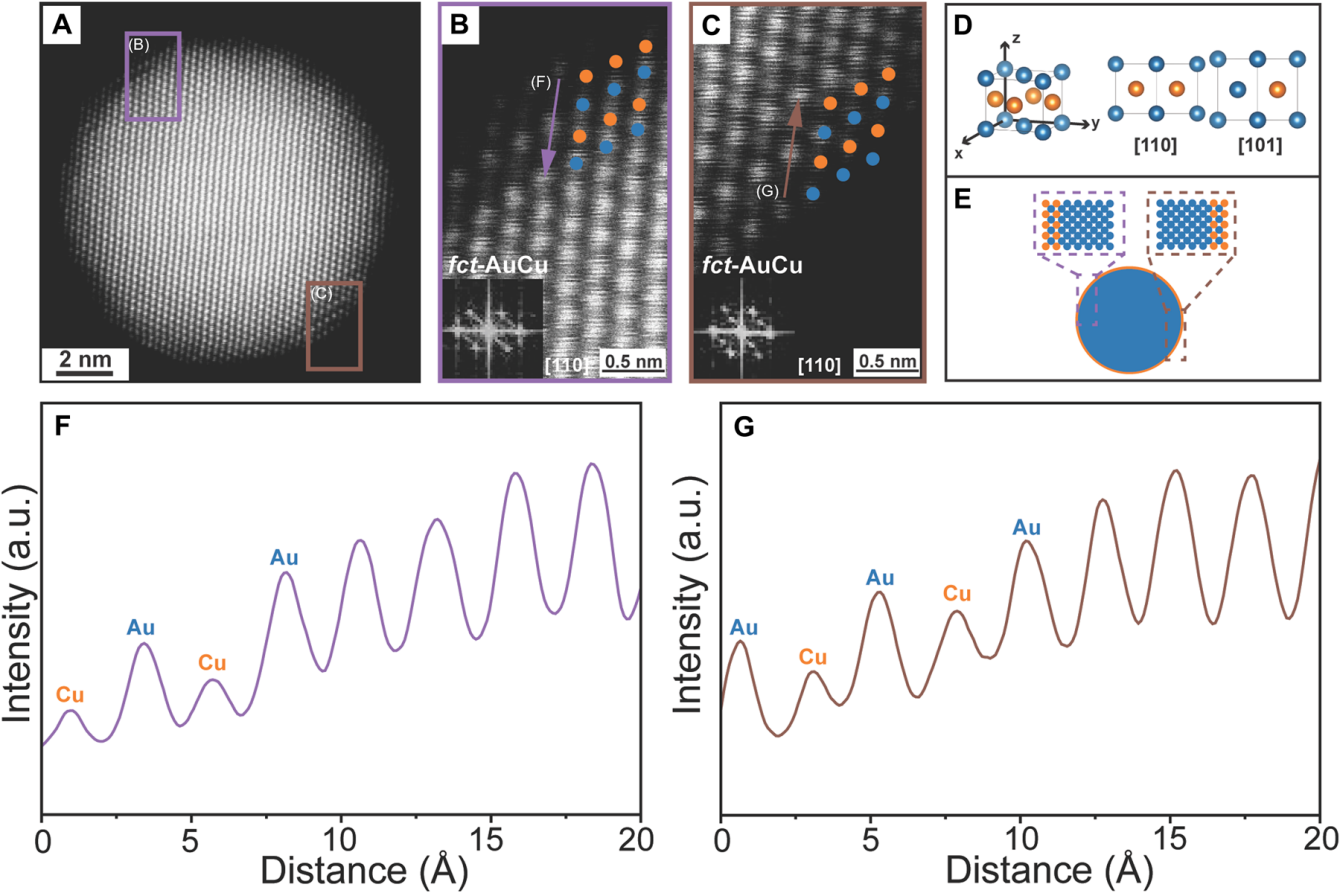
Characterizations of the x3 (1 hour) sample. (**A**) HAADF-STEM image along the [110] zone axis; (**B**) magnified HAADF-STEM image, with the inset showing an FFT pattern of the left side of the shell; (**C**) magnified HAADF-STEM image, with the inset showing an FFT pattern of the right side of the shell; (**D**) atomic model of an *fct*-AuCu lattice, together with the atomic pattern viewed along [110] and [101] directions; (**E**) schematic illustration of the atomic arrangements of the particles in the x3 (1 hour) sample; (**F** and **G**) atomic intensity profiles along purple and brown arrows in (B) and (C), respectively, showing the ordered arrangement of Au and Cu atoms on the (F) left side and (G) right side of the shell.

The results from TEM, EDS line-scanning, and UV-vis spectroscopy analyses collectively suggested that Cu was initially deposited on Au nanospheres in a symmetric manner, forming a uniform, ultrathin shell made of *fct*-AuCu intermetallic phase. The deposition was then switched to an asymmetric mode while converting part of the intermetallic phase into an *fcc*-Au_x_Cu_y_ alloy. As a result, a unique Janus shell was formed. We used HAADF-STEM to directly resolve the ultrathin shell on the particles in the x3 (1 hour) sample (fig. S10). Figure 5A shows the HAADF-STEM image of an individual particle along the [110] direction. The particle exhibited ordered Au and Cu atomic layers at two opposite regions (Fig. 5, B and C). The alternating Au-Cu atomic patterns matched well with the theoretical model of *fct-*AuCu intermetallic phase along [110], which was the zone axis for STEM (Fig. 5D). The agreement confirmed that the shell contained *fct*-AuCu phase on both sides of the particle (Fig. 5E). In addition, the atomic intensity profiles along the purple and brown arrows in Fig. 5, B and C, exhibited a periodic and alternating pattern of Au and Cu atoms (Fig. 5, F and G). Again, the STEM analysis validated that no Janus shell had been formed at this stage due to the involvement of uniform deposition of Cu. Taken together, the formation of this Au-Cu bimetallic nanocrystals with a unusual Janus shell can be divided into two major stages: (i) symmetric deposition of Cu on the entire surface of a Au seed, followed by Au-Cu inter-diffusion to form a uniform shell made of *fct*-AuCu intermetallic phase and (ii) preferential deposition of Cu on one side of the particle, accompanied by the transformation of the underlying intermetallic phase into an *fcc*-Au_x_Cu_y_ alloy.

We believe that the surface ligand should also play an important role in the synthesis of this class of unusual Au-Cu bimetallic nanocrystals. As documented in the literature, hexadecylamine (HDA) is essential to the elimination of oxidation for the newly deposited Cu atoms, helping stabilize the *fct*-AuCu intermetallic phase or *fcc*-Au_x_Cu_y_ alloy (*28*). Meanwhile, halide ions from the surface-bound cetyltrimethylammonium bromide/chloride (CTAB/C) have been reported to partially oxidize metal atoms on the surface, destabilizing the freshly-formed Cu atoms (*49*). Their difference in terms of presence or absence of halide ions allowed us to use x-ray photoelectron spectroscopy (XPS) to identify the surface ligands binding to the surface of the Au-Cu bimetallic nanocrystals (*50*–*52*). According to the XPS spectra shown in fig. S11, the CTAB/C was substituted by HDA during the deposition of Cu. Consequently, the freshly-deposited Cu atoms were stabilized against oxidation. The Cu 2p XPS spectra (fig. S12) further confirmed that the Cu was predominantly in the metallic state (Cu(0)), with only a minor contribution from Cu(II) species. Quantitative analysis based on the corresponding peak areas indicated that approximately 92% of the Cu was presented as Cu(0), with the other 8% corresponding to Cu(II).

### Catalytic performance toward electrochemical NO_3_RR

**Fig. 6.**
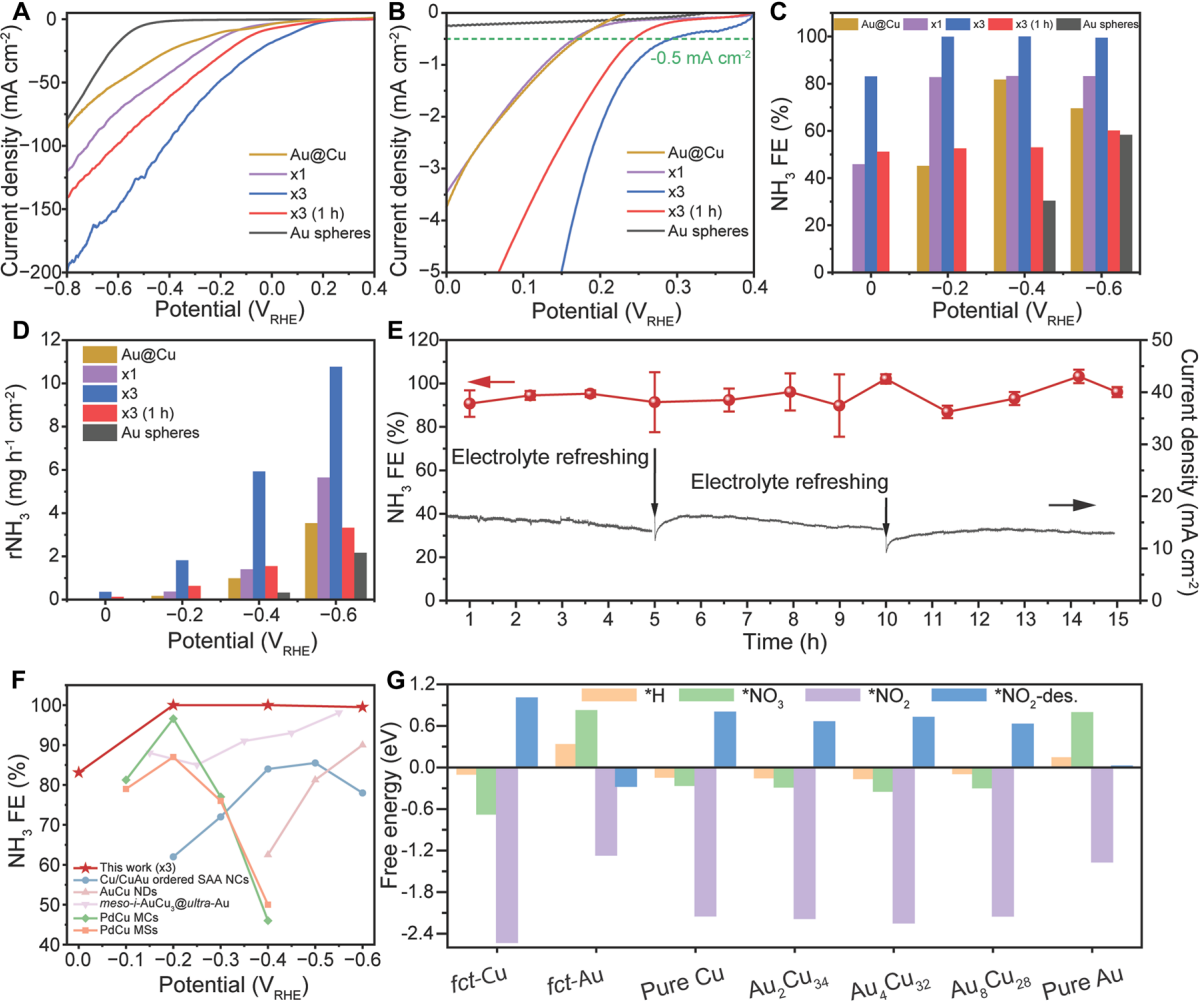
Electrochemical NO_3_RR performance. (**A**) LSV curves of the catalysts based on the Au@Cu, x1, x3, x3 (1 hour), and Au spheres in 1 M aqueous KOH containing 100 mM KNO_3_; (**B**) a segment of the LSV curves from 0 to 0.4 V_RHE_; (**C**) NH_3_ FE values; (**D**) NH_3_ yield rates; (**E**) potentiostatic stability measurement of the x3 sample at −0.2 V_RHE_ and error bars from independent samples; (**F**) comparison of NH_3_ FE values and applied potentials of the x3 sample with other state-of-the-art Cu-based electrocatalysts; (**G**) Calculated adsorption free energies of *H, *NO_3_, and *NO_2_, and desorption free energy of *NO_2_ on *fct*-AuCu, pure Cu, *fcc*-Au_x_Cu_y_ alloys, and pure Au.

To assess the merits of the Au-Cu bimetallic nanocrystals toward electrochemical NO_3_RR, the particles were dispersed on carbon black (fig. S13) and evaluated in a conventional three-electrode H-type cell (see Materials and Methods). As shown by the linear scanning voltammetry (LSV) curves, upon addition of 100 mM aqueous KNO_3_ into the electrolyte, all the catalysts exhibited higher current densities relative to that recorded in the plain electrolyte, indicating the occurrence of NO_3_RR (fig. S14 and Fig. 6A). Among them, the catalyst based on the x3 sample displayed the highest current density, implying its superior performance toward NO_3_RR. This catalyst also exhibited the lowest overpotential (η), with a value of only 397 mV, indicating the lowest energy barrier to NO_3_RR (Fig. 6B) (*53*).

Chronoamperometry tests were then performed across the potential range of 0 to −0.6 V_RHE_ (Fig. 6, C and D, and fig. S15). The concentrations of the main product (ammonia) and byproduct (nitrite) were quantified using conventional colorimetric methods (fig. S16). It should be noted that for the catalysts based on Au@Cu nanocrystals and Au nanospheres from 0 to −0.2 V_RHE_, the products could not be accurately determined due to the low current densities (<1 mA cm^−2^). Again, the x3 sample exhibited the highest selectivity toward NH_3_ among all the catalysts, achieving a Faradaic efficiency (FE) of 83.1% at 0 V_RHE_ and approximately 100% from −0.2 to −0.6 V_RHE_ (Fig. 6C). In addition, the x3 sample demonstrated the highest NH_3_ yield rates across the entire potential range (Fig. 6D). Stability tests conducted at −0.2 V_RHE_ over 15 hours indicated that both the NH_3_ FE value and current density were retained for the x3 sample (Fig. 6E). Moreover, post-reaction characterizations confirmed that the morphology and crystal structure were largely preserved during the stability test (figs. S17 and S18). Impressively, the x3 sample surpassed the performance of many Cu-based catalysts reported over the past five years for electrochemical NO_3_RR (Fig. 6F and table S2) (*34*, *54*–*57*).

To verify that the detected NH_3_ originated from NO_3_^−^ rather than nitrogen contamination, experiments involving isotope labeling and proton nuclear magnetic resonance (^1^H NMR) were performed (fig. S19). Given the wide variability in nitrate concentrations in actual wastewater sources (*55*), the catalytic performance of the x3 sample toward NO_3_RR was evaluated across a broad range of nitrate concentration from 10 to 1000 mM (fig. S20). As expected, the NH_3_ yield rate increased with increasing nitrate concentration. In addition, the NH_3_ FE exceeded 90% across the broad range of concentration and remained as high as 86.1% even at a low nitrate concentration of 10 mM, highlighting the promise of the x3 sample.

To investigate the underlying factors contributing to the superior NO_3_RR performance of the x3 sample, a series of systematic studies was conducted. In the x3 sample, the electrochemically active surface area (ECSA) was maximal among all the catalysts (fig. S21 and table S3). We also measured the FE and yield rates for nitrite (NO_2_^−^) (fig. S22), the critical intermediate during NO_3_RR. The result suggested that the x3(1 hour) sample exhibited relatively high FE and the highest yield rates toward NO_2_^−^ at all the measured potentials, indicating that the surface made of *fct*-AuCu intermetallic phase promoted the generation of NO_2_^−^. Given the essential role of active ^*^H in the hydrogenation of NO_2_^−^, the generation and consumption of active ^*^H during electrocatalysis were further examined. As shown in fig. S23, in the absence of NO_3_^−^, the x3 sample exhibited a large oxidation peak area of active ^*^H, indicating efficient water dissociation. Upon the introduction of NO_3_^−^, the oxidation peak area of active ^*^H decreased, suggesting its consumption for the electrosynthesis of NH_3_. The higher-potential peak corresponded to surface Cu oxidation, which was suppressed in the presence of NO_3_^−^, likely due to competitive adsorption of intermediates on Cu sites that limited Cu-O formation. Notably, this oxidation feature arose during the anodic sweep in cyclic voltammetry (CV), while under the negative potentials used for NO_3_RR, Cu remained predominantly in the metallic state, consistent with the reversible Cu redox behavior observed in the CV profiles.

To gain mechanistic insight into the distinct roles of *fct*-AuCu intermetallic and *fcc*-Au_x_Cu_y_ alloy phases, we performed density functional theory (DFT) calculations to evaluate the adsorption free energies of *H, *NO_3_ and *NO_2_, as well as the desorption free energy of *NO_2_ (denoted *NO_2_-des.), as shown in Fig. 6G. These descriptors were selected as they corresponded to the critical elementary steps in the reaction pathway, namely hydrogen adsorption (*H), nitrate adsorption and activation (*NO_3_), intermediate stabilization (*NO_2_), and the release of NO_2_^−^ into the electrolyte (NO_2_-des.). To ensure the relevance of the theoretical models to the experimental system, multiple surface configurations were considered (fig. S24). For the *fct*-AuCu intermetallic phase, both Cu-terminated (*fct*-Cu) and Au-terminated (*fct*-Au) surfaces were constructed, as both terminations were experimentally observed in the HAADF-STEM image of the x3 (1 hour) sample (Fig. 5, B and C). For the *fcc*-Au_x_Cu_y_ alloys, a series of compositions (Au_2_Cu_34_, Au_4_Cu_32_ and Au_8_Cu_28_) were modeled to represent different Au to Cu ratios, covering distinct samples and enabling systematic investigation of the composition-dependent adsorption behavior. In addition, pure Cu and pure Au surfaces were included as reference systems to benchmark the intrinsic adsorption characteristics. The optimized adsorption configurations and corresponding Bader charge analyses are provided in fig. S25.

The calculated results revealed several notable trends. Pure Au exhibited a highly positive adsorption free energy for *NO_3_, indicating weak interaction with nitrate species. This was consistent with its more negative onset potential and limited activity toward nitrate reduction. In contrast, Cu-containing surfaces effectively stabilized *NO_2_, suggesting their ability to facilitate nitrate activation and intermediate formation. More importantly, the *fct*-Cu surface exhibited more favorable *NO_3_ adsorption, facilitating the initial activation of nitrate. In contrast, the *fct*-Au surface showed a relatively low desorption free energy for *NO_2_, indicating that once formed, *NO_2_ could be readily released into the electrolyte. At the same time, *H adsorption on *fct*-Au was comparatively less favorable than on *fcc*-Au_x_Cu_y_ alloys, suggesting a weaker tendency for subsequent hydrogenation. Therefore, the coexistence of Cu- and Au-terminated surfaces in the *fct*-AuCu phase enabled a synergistic mechanism, by which NO_3_^−^ activation preferentially occurred on Cu sites, while the generated NO_2_^−^ intermediate could readily desorb from adjacent Au sites. This spatial and functional decoupling of activation and desorption steps effectively promoted NO_2_^−^ formation while suppressing its further hydrogenation, thereby explaining the experimentally observed high NO_2_^−^ production rate for the x3 (1 hour) catalyst. In contrast, the *fcc*-Au_x_Cu_y_ alloy exhibited more favorable *H adsorption, promoting the hydrogenation of NO_2_^−^ to downstream products such as NH_3_. As a result, NO_2_^−^ was less accumulated on the surface, leading to a relatively lower NO_2_^−^ FE or yield. Furthermore, the adsorption energetics displayed a volcano-type dependence on the Au to Cu ratio. Both excessively low and excessively high Cu contents were unfavorable for the adsorption of key species such as *H and *NO_3_. The optimized composition in the x3 catalyst provided a balanced adsorption strength, enabling efficient NO_3_^−^ activation while maintaining appropriate *H adsorption and intermediate turnover.

Integrating these observations, we conclude that the atomic ratio and arrangement of Au and Cu both have substantial impacts on the NO_3_RR activity. Specifically, the *fct*-AuCu intermetallic phase promotes the generation of intermediate NO_2_^−^, whereas the *fcc*-Au_x_Cu_y_ alloy enhances *H adsorption. The x3 sample, which uniquely integrates intermetallic and alloy phases side by side on the surface, results in outstanding electrocatalytic performance toward NO_3_RR.

## DISCUSSION

Previous research suggested that Janus nanocrystals exist in a side-by-side configuration of two distinct metals, while their atomic structures and correlation with electrocatalytic performance remain unclear. In this work, we have elucidated the atomic details involved in the synthesis of Au-Cu bimetallic nanocrystals with an unusual Janus surface that features two distinct bimetallic structures—an intermetallic phase and an alloy—on the surface of each particle. The formation of this unique Janus surface involves two distinct stages. In the initial stage, a uniform, ultrathin shell made of the *fct*-AuCu intermetallic phase is formed due to uniform and symmetric deposition of Cu atoms and their subsequent inter-diffusion with the underlying Au core. As the reaction proceeds, the growth is switched to an asymmetrical mode. The limited supply of Cu atoms, together with strain accumulation during shell thickening, can induce preferential growth at the activated site on the shell surface. As a result, the intermetallic phase on the side with more Cu deposition evolves into an *fcc*-Au_x_Cu_y_ alloy, generating the unique Janus surface. Although the present study focuses on Cu growth on Au, the two-stage growth involving the formation of an ultrathin and complete shell, followed by symmetry breaking, may be broadly applicable to other systems. While the specific details, such as formation of intermetallic and alloy phases, are dependent on the metals involved, the involvement of symmetric shell growth followed by transition to asymmetric growth mode may represent a universal phenomenon that has been overlooked and missed in the literature. When employed as electrocatalysts toward the NO_3_RR, the Janus nanocrystals exhibit FE values near 100% across a wide potential window from −0.2 to −0.6 V_RHE_, highlighting their great potential for advanced electrocatalytic applications. This study not only expands the structural diversity of Janus nanocrystals but also provides insights into growth mechanisms for tailoring Janus architectures with enhanced electrocatalytic performance.

## MATERIALS AND METHODS

### Chemicals and materials

Gold(III) chloride trihydrate (HAuCl_4_·3H_2_O, ≥99.9%), sodium borohydride (NaBH_4_, 98%), ascorbic acid (AA, ≥99%), cetyltrimethylammonium bromide (CTAB, ≥99%), and cetyltrimethylammonium chloride (CTAC, 25 wt.% in water), hexadecylamine (HDA, ≥98%), copper(II) chloride dihydrate (CuCl_2_·2H_2_O, ≥99%), D-(+)-glucose (≥99.5%), potassium hydroxide (KOH, ≥85%), potassium nitrate (KNO_3_, ≥99%), potassium nitrate (KNO_3_-^15^N, 98 atom% ^15^N), potassium nitrite (KNO_2_, ACS, ≥97%), ammonium chloride (NH_4_Cl, 99.5%), sulfanilamide (C_6_H_8_N_2_O_2_S, ≥98%), N-(1-Naphthyl)ethylenediamine dihydrochloride (C_12_H_14_N_2_, ≥98%), potassium sodium tartrate tetrahydrate (KNaC_4_H_4_O_6_·4H_2_O, 99%), mercury(II) iodide red (HgI_2_, ≥99%), sodium hydroxide (NaOH, ≥97%), and potassium iodide (KI, ≥99%) were all obtained from Sigma-Aldrich and used as received. Deionized (DI) water with a resistivity of 18.2 MΩ·cm at room temperature was used throughout the experiments.

### Synthesis of CTAB/C-capped Au nanospheres

We followed a published protocol to synthesize CTAB/C-capped Au spheres of 10 nm in diameter. In the first step, Au clusters were prepared by mixing 5 ml of aqueous CTAB (200 mM) and 5 ml of aqueous HAuCl_4_ (0.5 mM) in a 20-ml glass vial, followed by the introduction of 0.6 ml of freshly prepared aqueous NaBH_4_ (10 mM) in one shot at 27°C. The mixture was placed on an orbital shaker set to a speed of 270 rpm for 2 min and then kept undisturbed at 27°C for at least 3 hours to allow the remaining NaBH_4_ to completely decompose. Meanwhile, 2 ml of aqueous CTAC (200 mM) and 2 ml of aqueous HAuCl_4_ (0.5 mM) were mixed in a separate 20-ml glass vial, followed by one-shot injection of 1.5 ml of aqueous AA (100 mM) under magnetic stirring (600 rpm) and at 27°C. Then, 50 μl of the just-obtained Au clusters was introduced in one shot and the reaction was allowed to proceed for 15 min. The solid products were collected by centrifugation at 14500 rpm for 30 min and washed once with water. Afterwards, the particles were re-dispersed in 1 ml of aqueous CTAC (20 mM) and used as seeds for the growth of Cu.

### Synthesis of the Au-Cu bimetallic nanocrystals

In a 20-ml glass vial, 45 mg (207.07 μmol) of HDA was mixed with 2 ml of water. Due to the poor solubility of HDA in water at room temperature, the mixture was ultrasonicated for 10 min to improve the dispersion of HDA in water. Two solutions containing 138.77 mM glucose and 3.44 mM CuCl_2_ were prepared, respectively, by dissolving 50 mg (277.54 μmol) of glucose in 2 ml of water and 0.53 mg (3.08 μmol) of CuCl_2_·2H_2_O in 1 ml of water. Next, 2 ml of the aqueous glucose and 1 ml of the aqueous CuCl_2_ were sequentially added into the HDA dispersion. Afterwards, 200 μl of the aqueous suspension containing the 10-nm Au spherical seeds at a specific concentration (see table S1 for details) was added. Glucose served as a mild reducing agent for Cu(II) precursor and HDA was used as a coordination ligand for Cu(II) and a capping agent for passivating the newly-formed Cu surface. Due to the difference in binding strength, HDA could also replace the original CTAB/C on the Au surface. The vial was placed in an oil bath held at 27°C at a stirring rate of 280 rpm for 12 hours to obtain a homogenous mixture with a milky pink color. To remove the oxygen trapped in the vial, Ar was blown over the mixture for 10 min before the vial was tightly capped, transferred into an oil bath set to 100°C, and heated for 5 hours under magnetic stirring at 280 rpm. The reaction was quenched by letting the vial cool to room temperature in about 5 min. Then, the reddish-brown mixture was transferred into a 50-ml centrifuge tube and water was added until the total volume reached 30 ml. The solid products were collected by centrifugation for 10 min at a speed of 9835 rpm and then washed twice with 1 ml of water to remove the excess HDA by centrifugation at 9835 rpm for another 10 min. After extracting the supernatant, the solid was re-dispersed in 1 ml of water for further characterizations.

For the synthesis of Au@Cu nanocrystals, except increasing the amount of CuCl_2_·2H_2_O to 10.5 mg (61.6 μmol) dissolved in 1 ml of water, all other parameters were kept the same as the preparation of the x1 sample.

### Characterizations

Hitachi HT7700 microscope was used at 120 kV to take transmission electron microscopy (TEM) images. Ultraviolet-visible (UV-vis) spectra were recorded on a Cary 60 spectrometer (Agilent Technologies, Santa Clara, CA). The concentration of Au nanospheres was determined using an inductively-coupled plasma mass spectrometer (ICP-MS, NexION 300Q, PerkinElmer, Waltham, MA), together with the size measured from TEM images. High-resolution transmission electron microscopy (HRTEM), energy-dispersive x-ray spectroscopy (EDS) mapping, and line-scanning analyses were conducted using an FEI Tecnai G2 F30 TEM operated at 200 kV. High-angle annular dark-field scanning electron microscopy (HAADF-STEM) images were taken on a Hitachi HD-2700 microscope at 200 kV. X-ray photoelectron spectroscopy (XPS) analyses were conducted using Thermo K-Alpha XPS. X-ray diffraction (XRD) patterns were obtained using Rigaku Miniflex Powder XRD. Proton nuclear magnetic resonance (^1^H NMR) spectra were obtained using a Bruker AV3 HD 500 MHz NMR spectrometer.

### Preparation of carbon-supported catalysts (20% loading)

To prepare carbon-supported catalysts, we mixed an aqueous dispersion of 0.2 mg of catalysts (quantified by ICP-MS) with 0.8 mg activated carbon (Vulcan XC-72R) and sonicated for 1 hour. The catalysts were collected by centrifugation, washed three times with excess ethanol, and redispersed in 200 μl ethanol.

### Electrochemical measurements

The electrochemical nitrate reduction reaction (NO_3_RR) was performed on an electrochemical workstation (CHI 600E) with a three-electrode system in a standard H-type cell at room temperature. A graphite rod and Hg/HgO (1 M KOH) were used as counter and reference electrodes, respectively. First, 4 μl of Nafion solution (5 wt%, Sigma-Aldrich) was added to 200 μl of ethanol mentioned above, and the mixture was then ultrasonicated for at least 30 min to generate a homogeneous ink. Next, the dispersion was transferred onto 1 cm^2^ carbon paper (Sigracet 28 BC), leading to a metal loading of ∼0.2 mg cm^−2^. An aqueous solution comprising 1 M KOH was utilized as the anodic electrolyte, while a mixture of 1 M KOH and 100 mM KNO_3_ served as the cathodic electrolyte. The anodic and cathodic compartments were separated by an anion-exchange membrane (Fumasep FAA-3-50). Before the electrochemical measurement, the cathodic electrolyte was degassed with Ar gas for 30 min to eliminate any potential interference from nitrogen. In addition, all samples were preconditioned by potential cycling in 1 M aqueous KOH until stable polarization curves were obtained. This treatment ensured the removal of residual surfactants and surface-adsorbed impurities. All the potentials in this study were referenced to Hg/HgO (measured) or the RHE. The potential was converted to RHE according to *E* (vs. RHE) = *E* (vs. Hg/HgO) + 0.098 V + 0.059 × pH. Linear sweep voltammetry (LSV) and chronoamperometry measurements were conducted, respectively, to qualitatively and quantitatively assess the NO_3_RR performance. After completion of each 60-minute chronoamperometry test, the concentrations of ammonia and nitrite in the electrolytes were determined via chromogenic methods. Cyclic voltammetry (CV) measurements taken with various scan rates (20, 40, 60, 80, and 100 mV s^−1^) were conducted in static solution to estimate the double-layer capacitance (C_dl_) by sweeping the potential across the non-Faradaic region −0.05 to 0.05 V_Hg/HgO_. The electrochemically active surface area (ECSA) was subsequently calculated by normalizing C_dl_ to a specific capacitance value (C_s_) of 0.04 mF cm^−2^. The stability test was conducted at −0.2 V_RHE_ for 15 hours. The overpotential (η) was calculated: η = 0.69 V − *E*, where *E* is the potential at a current density of −0.50 mA cm^−2^.

### Quantification of NH_3_

Preparation of colorant: Dissolve 16.0 g NaOH in 50 ml of water to obtain solution A. Dissolve 7.0 g KI and 10.0 g HgI_2_ in 25 ml of water to obtain solution B. Mix solution A and B under vigorous stirring and then volume in a 100 ml volumetric flask. Preparation of potassium sodium tartrate solution: Dissolve 100.0 g of potassium sodium tartrate in 200 ml of water. First, take an appropriate amount of electrolyte and dilute it to the detection range. Add 0.1 ml of potassium sodium tartrate solution and 0.1 ml of colorant to 5 ml of diluted electrolyte. After shaking and standing for 30 min, the absorbance was measured at 420 nm. The ammonia standard curve was obtained by measuring different concentrations of NH_4_Cl solution.

### Quantification of NO_2_^−^

Preparation of colorant: Dissolve 20.0 g sulfanilamide and 1.0 g N-(1-Naphthyl)ethylenediamine dihydrochloride in a mixture of 250 ml of water and 50 ml of phosphoric acid, and then volume in a 500 ml volumetric flask. First, take an appropriate amount of electrolyte and dilute it to the detection range. Add 0.1 ml of colorant to 5 ml of diluted electrolyte. After shaking and standing for 30 min, the absorbance was measured at 540 nm. The nitrite standard curve was obtained by measuring different concentrations of KNO_2_ solution.

### Isotope labeling experiments

The KNO_3_-^15^N (98 atom%) was used as the feeding N-source for the isotopic labeling of NO_3_RR to elucidate the source of NH_3_. After electroreduction, a certain amount of electrolyte was taken out for further quantification by ^1^H NMR.

## Supplementary Materials

### This PDF file includes:

Supplementary Text Figs. S1 to S26 Tables S1 to S3 References
