# Supplementary material for: What is happening at the atomic level during the seed-mediated growth of copper on gold?
Source: Sci Adv. 2026 Aug 5;12(32):eaee8346. doi: 10.1126/sciadv.aee8346 (PMC13440395; doi:10.1126/sciadv.aee8346)
Supplement: Supplementary file 1 [file sciadv.aee8346_sm.pdf]

Science Advances
AAAS

Supplementary Materials for

What is happening at the atomic level during the seed-mediated growth of copper on gold?

Yanzi Yan *et al.*

Corresponding author: Younan Xia, younan.xia@bme.gatech.edu

Sci. Adv. **12**, eaee8346 (2026)
DOI: 10.1126/sciadv.aee8346

This PDF file includes:

Supplementary Text
Figs. S1 to S26
Tables S1 to S3
References

Supplementary Text

Computational methods

Density functional theory (DFT) calculations for electrochemical reactions were performed *via* the QUICKSTEP module of CP2K 2025.1 package (*58*). Firstly, bulk structures of *fcc*-Au, *fcc*-Cu and *fct*-AuCu were optimized for both lattice constants and atom coordinates at PBEsol/DZVP-MOLOPT-SR-GTH level (*59*, *60*). Then a 3×3×1 super cell of Cu bulk was constructed and 2, 4 or 8 Cu atoms were replaced by Au atoms respectively. The substitutional sites were rationally selected to maximize the Au-Au distance. Next these bulk structures of Au-Cu alloy were optimized to release the internal stress introduced by Au atoms. Finally, four-layer slab models of (100) orientation with a c-axis of 30 Å to eliminate the interaction from periodic images were built, and different intermediates (*H, $*NO_3$ and $*NO_2$) were adsorbed on these slabs and optimized at PBE-D3(BJ)/DZVP-MOLOPT-SR-GTH level (*61*, *62*) with the bottom two layers fixed to present properties of bulk phase. The adsorption sites were also rationally selected to obtain the most stable configuration (*34*, *63*). To obtain more accurate electronic energies, single point calculations were performed at PBE-D3(BJ)/TZV2P-MOLOPT-PBE-GTH level based on the optimized structures. During optimization, the convergence thresholds of self-consistent field (SCF) and maximum force were chosen as 1×10^{-6} Hartree and 4.5×10^{-4} Hartree/Bohr respectively. Besides, the energy cutoff was set as 600 Ry, and the Brillouin zone was sampled with a gamma-centered Monkhorst-Pack k-point mesh as follows: *fcc*-Au: 10×10×10; *fcc*-Cu: 12×12×12; *fct*-AuCu: 15×15×12; slabs: 3×3×1. The Fermi-Dirac smearing algorithm with an electronic temperature of 300 K was used to accelerate convergence, and the contribution from electronic entropy was eliminated as mentioned in reference (*64*). The visualization of optimized structures and the calculation of Bader charges were realized *via* VESTA (*65*) and the Bader (*66*) program respectively.

Gibbs free energy change ΔG of electrochemical reactions was obtained by the computational hydrogen electrode (CHE) model (*67*), and the details were as follows.

For

$$* + e^- + H^+ \rightarrow *H \quad (1)$$

Since

$$G_{e^-} + G_{H^+} = \frac{1}{2} G_{H_2} \quad (2)$$

Then

$$\Delta G_H = G_{*H} - G_* - \frac{1}{2} G_{H_2} \quad (3)$$

For

$$*NO_2 + e^- \rightarrow * + NO_2^- \quad (4)$$

Add H^+ to both sides of the equation

$$* + e^- + H^+ \rightarrow * + NO_2^- + H^+ \quad (5)$$

Since

$$G_{NO_2^-} + G_{H^+} = G_{HNO_2} + 0.20\ eV \quad (6)$$

Then

$$\Delta G_{NO_2,d} = G_* + G_{HNO_2} - G_{*NO_2} - \frac{1}{2}G_{H_2} + 0.20\ eV \quad (7)$$

0.20 eV was calculated from the standard free energy change of nitrous acid dissociation, i.e., calculated by $pK_a = 3.4$ at 298.15 K *via* $\Delta G^\theta = 2.303RTpK_a$.

For

$$* + NO_3^- + 2H^+ + e^- \rightarrow * NO_2 + H_2O \quad (8)$$

It can be transformed into

$$* + (NO_3^- + H^+) + (e^- + H^+) \rightarrow * NO_2 + H_2O \quad (9)$$

Since

$$G_{NO_3^-} + G_{H^+} = G_{HNO_3} - 0.083\ eV \quad (10)$$

Then

$$\Delta G_{NO_2,a} = G_{*NO_2} + G_{H_2O} - G_* - \frac{1}{2}G_{H_2} - G_{HNO_3} + 0.083\ eV \quad (11)$$

–0.083 eV was calculated from the standard free energy change of nitric acid dissociation, i.e., calculated by $pK_a = -1.4$ at 298.15 K *via* $\Delta G^\theta = 2.303RTpK_a$.

For

$$* + NO_3^- \rightarrow * NO_3 + e^- \quad (12)$$

Add H^+ to both sides of the equation

$$* + NO_3^- + H^+ \rightarrow * NO_3 + e^- + H^+ \quad (13)$$

Then

$$\Delta G_{NO_3} = G_{*NO_3} + \frac{1}{2}G_{H_2} - G_* - G_{HNO_3} + 0.083\ eV \quad (14)$$

The computation of Gibbs free energy G was as follows:

For pure slabs, it was assumed that $G \approx E$.

For isolated molecules, the Gibbs free energy was calculated by

$$G = E + \Delta G_{gas} + \Delta G_{sol} + RTln(\frac{C}{C_0}) \quad (15)$$

ΔG_{gas} was obtained by conducting vibrational analysis at 298.15 K and 101.325 kPa *via* Shermo 2.6.1 package (*68*).

$$\Delta G_{gas} = ZPE + \int C_P(T)dT - TS \quad (16)$$

ΔG_{sol} was obtained by either form reference (*69*) or DFT calculations (except for H_2).

$$\Delta G_{sol} = E_{sol} - E_{gas} \quad (17)$$

E_{sol} and E_{gas} were obtained at M05-2X/6-31G(d) level (*70, 71*) *via* ORCA 5.0.4 package (*72*) and SMD model (*73*) was utilized to present the effect of water environment.

$RTln(\frac{C}{C_0})$ represents the concentration correction from ideal gas to 1M solution (except for H_2), i.e., C_0 equals 0.040874 and C equals 1.

For slabs with absorbed intermediate, the Gibbs free energy was calculated by

$$G_{*A} = E_{*A} + \Delta G_{gas,*A} = E_{*A} + ZPE + \int C_P(T)dT - TS \quad (18)$$

It should be noted that when performing vibrational analysis for *A, metal atoms were fixed.

All experimental measurements were repeated at least three times to ensure reproducibility. Unless otherwise specified, all data represent technical replicates.

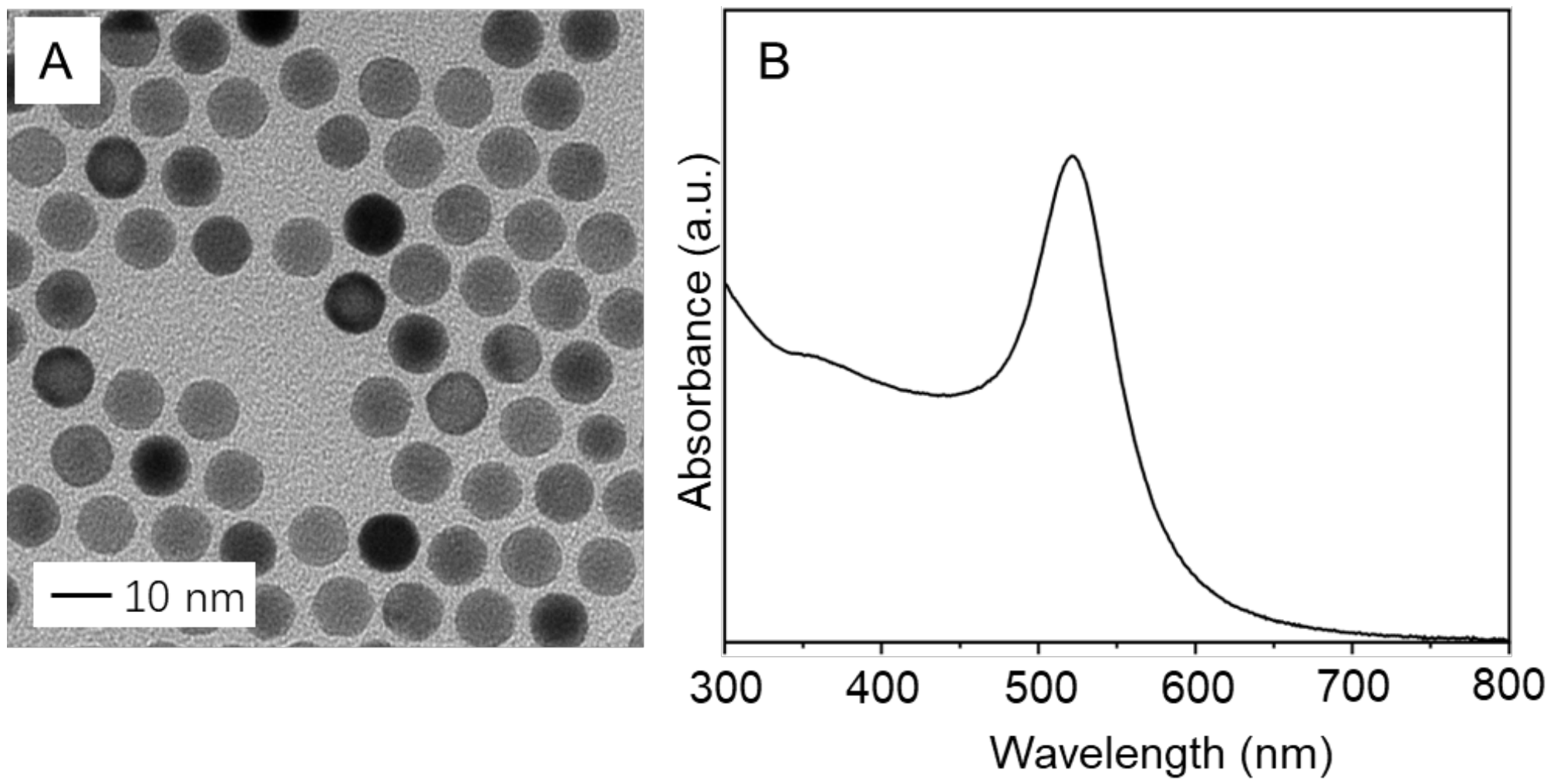

Fig. S1. (A) Transmission electron microscopy (TEM) image and (B) ultraviolet-visible (UV-vis) spectrum of the Au nanospheres serving as seeds for Cu deposition. The spectrum was recorded from an aqueous suspension of the nanospheres. The particles exhibited a sharp localized surface plasmon resonance (LSPR) peak at 521 nm in (B), indicating their high quality in terms of size uniformity and colloidal stability.

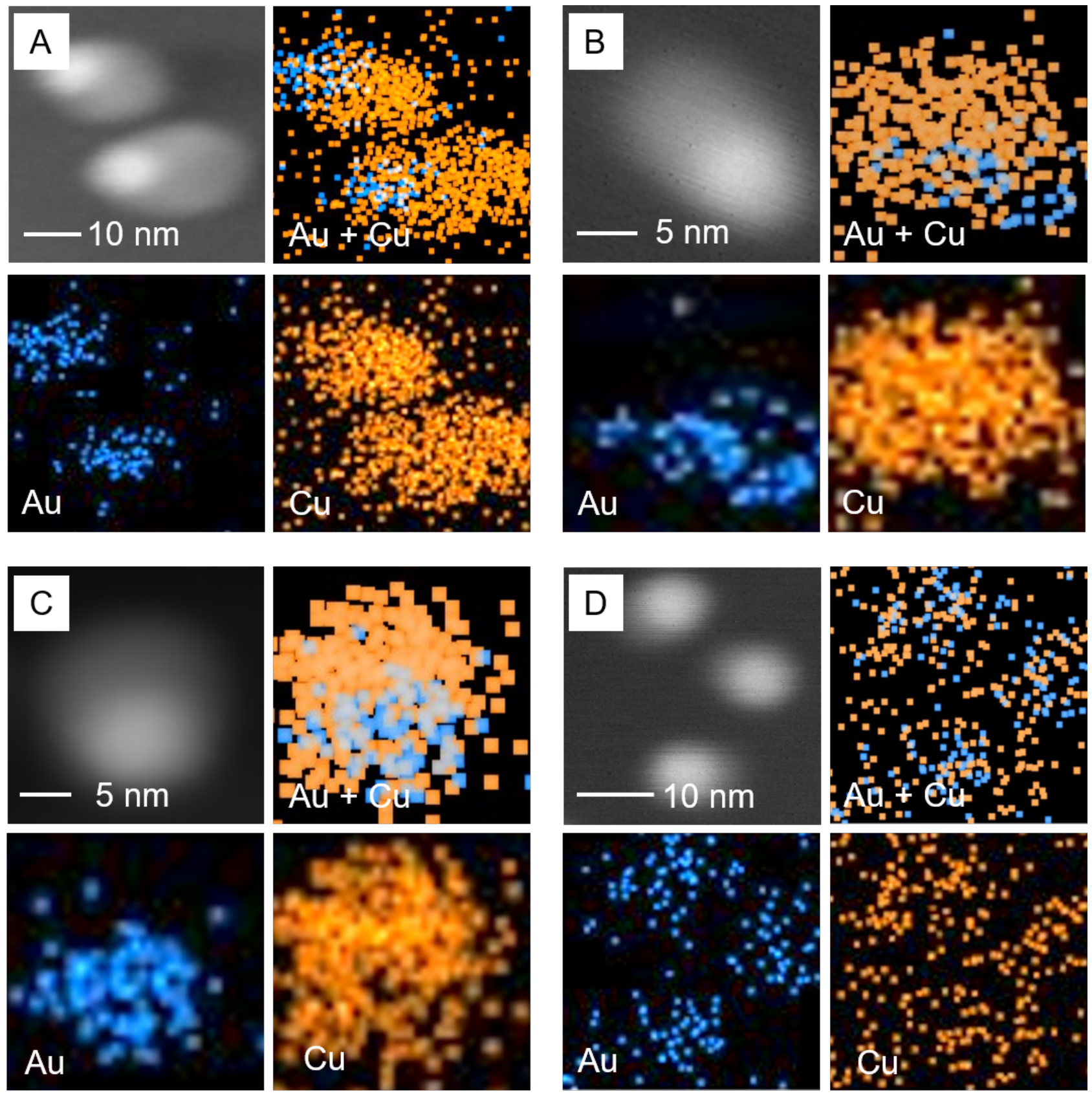

Fig. S2. Energy-dispersive X-ray spectroscopy (EDS) mapping of Au-Cu bimetallic nanocrystals synthesized with Au spherical seeds supplied at different folds relative to the standard protocol (3.11×10^{10}): (A) x1, (B) x2, (C) x3, and (D) x4. The small size of the particle involved in this work gave a high level of background noise, compromising the resolution of mapping.

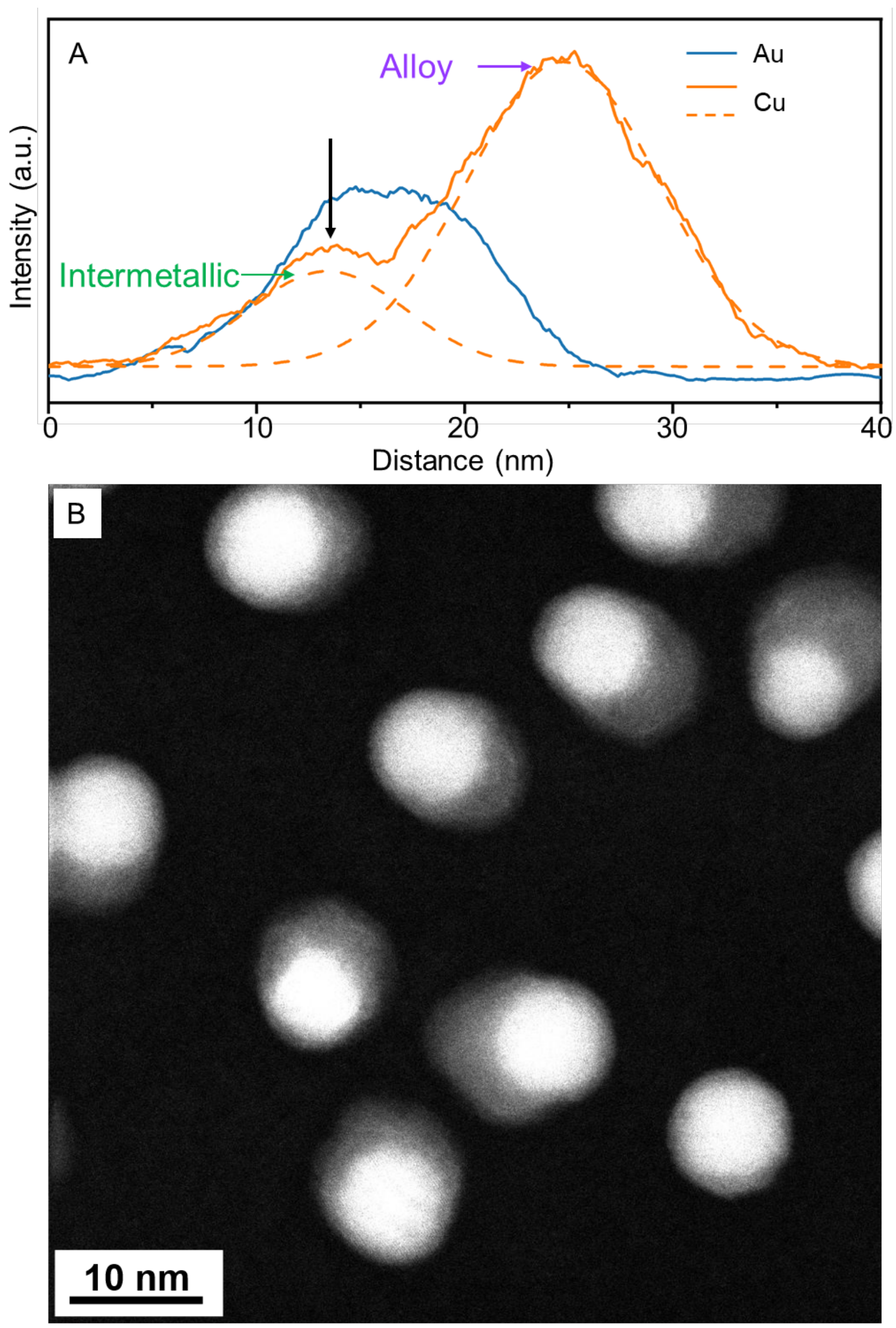

Fig. S3. (A) Deconvoluted EDS line-scanning of an individual particle in the x3 sample. This EDS line-scanning is the same as Fig. 2F, except the deconvolution of Cu signal as well as the indications of intermetallic and alloy. A small bump in Cu signal was observed in the EDS line-scanning profile as indicated by the black arrow. By deconvoluting the small bump from the major Cu signal, it is clear that this bump had a distance relative to the Au seed. The dot lines indicate the Cu in face-centered-tetragonal (*fct*)-AuCu intermetallic (green arrow) and face-centered-cubic (*fcc*)-Au_xCu_y alloy (purple arrow) phases of the particle. (B) High-angle annular dark-field scanning electron microscopy (HAADF-STEM) image of the corresponding sample.

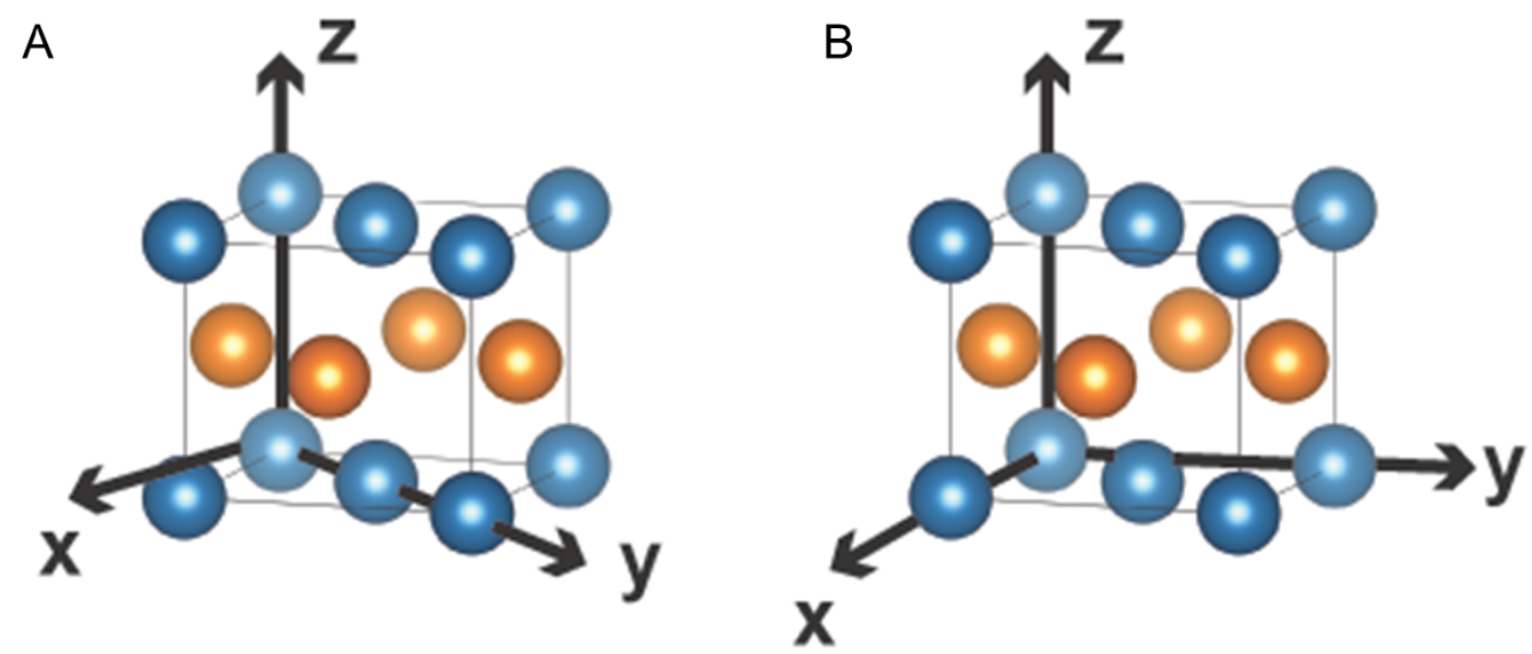

Fig. S4. (A) Conventional *fct* lattice cell and (B) the *fct* lattice cell used in this work. Note that the yx-plane in (B) is rotated by 45° relative to that in (A).

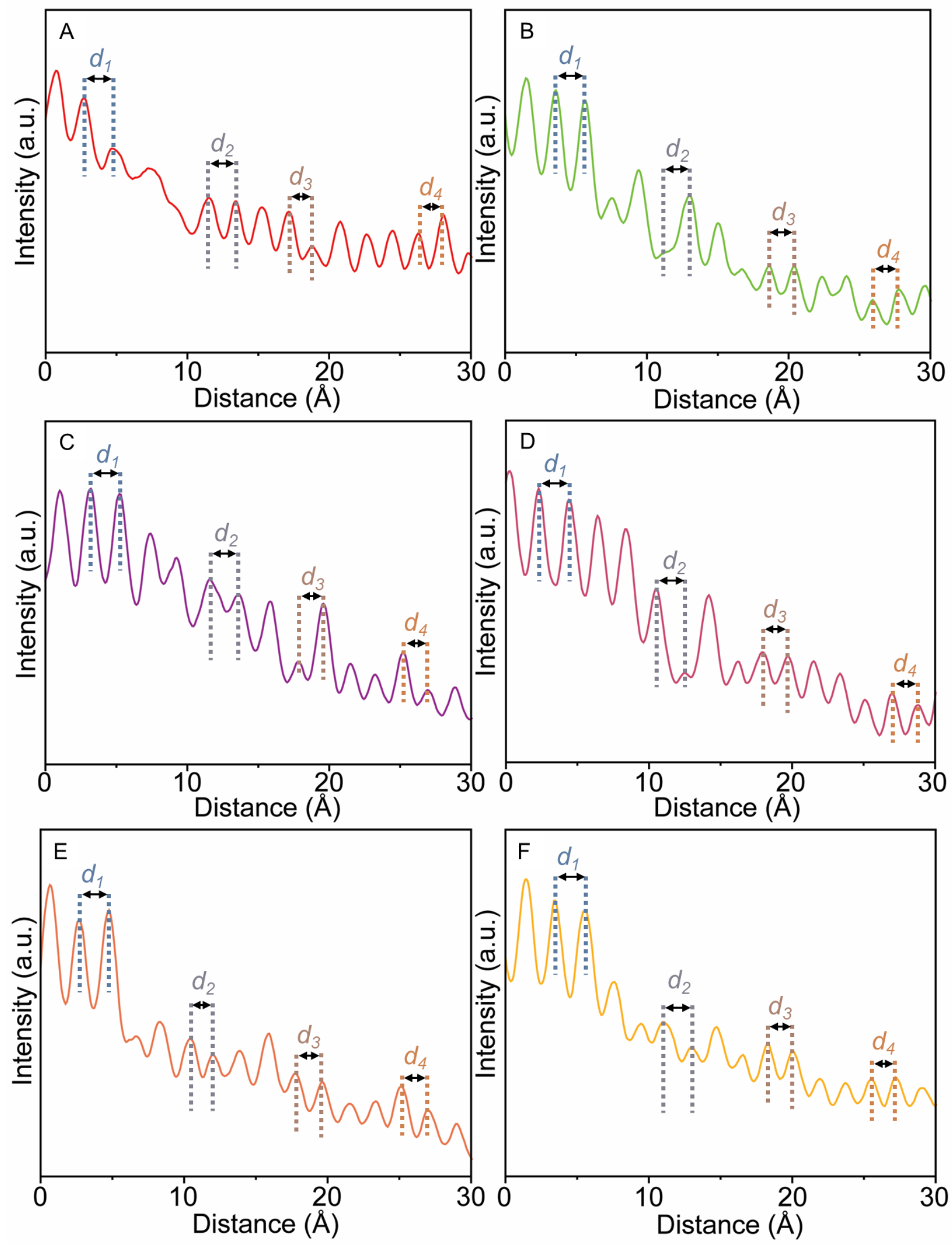

Fig. S5. Line profiles used for deriving the statistical ratio of Au to Cu in the alloy part of the x3 sample. We calculated the average $d_{(002)}$ at different locations on each line profile (A-F, corresponding to the six arrows from top to bottom in Fig. 3H) and compared the measured value with $d_{(002)}$ of pure Au and Cu using Vegard's Law (*35*) to obtain the Au to Cu atomic ratio. It should be noted that $d_{(002)}$ is equal to $d_{(200)}$ in *fcc*-Au_xCu_y alloy.

$$d_{(200)} \text{ in pure Au} = 2.09 \text{ Å}$$

$$d_{(200)} \text{ in pure Cu} = 1.79 \text{ Å}$$

$$\text{Au-Cu atomic ratio} = \frac{d\text{-}1.79}{2.09\text{-}d} \qquad (d = d_1, d_2, d_3, d_4)$$

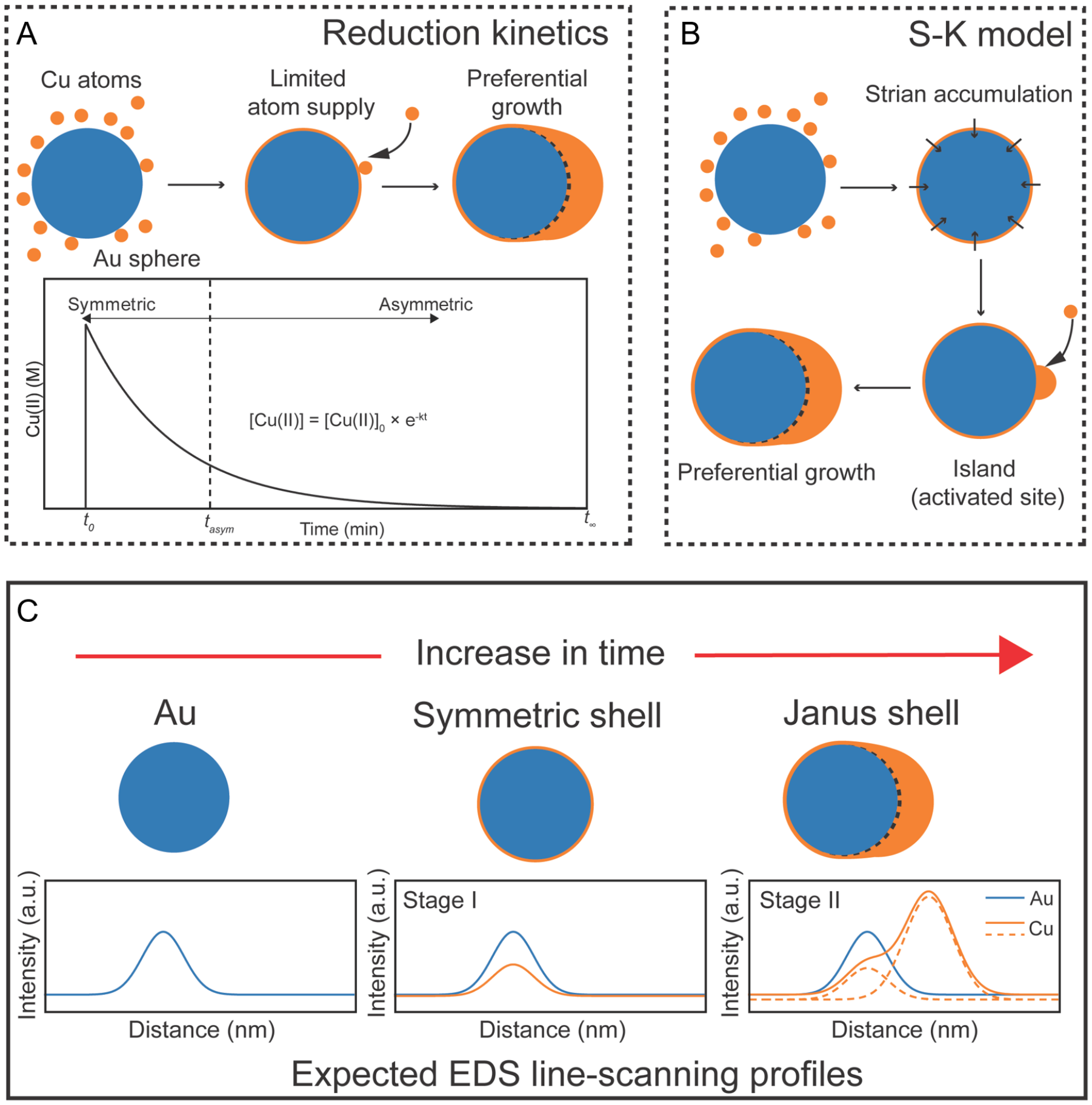

Fig. S6. Schematic illustrations of two possible mechanisms: (A) kinetics-controlled mechanism. In the synthesis, the Cu(II) precursor is reduced by glucose to generate Cu atoms, which then nucleate on the surface of the Au seeds. Since the glucose is present in large excess over the precursor, the reduction can be approximated as a pseudo-first-order reaction with respect to the precursor. The equation can be used to derive the instantaneous concentration of Cu(II) remaining in the reaction solution as a function of time. $[Cu(II)]_0$ corresponds to the initial concentration and *k* is the rate constant; (B) Stranski-Krastanov (S-K) model mechanism. (C) The expected EDS line-scanning profiles of an individual particle in the x3 sample.

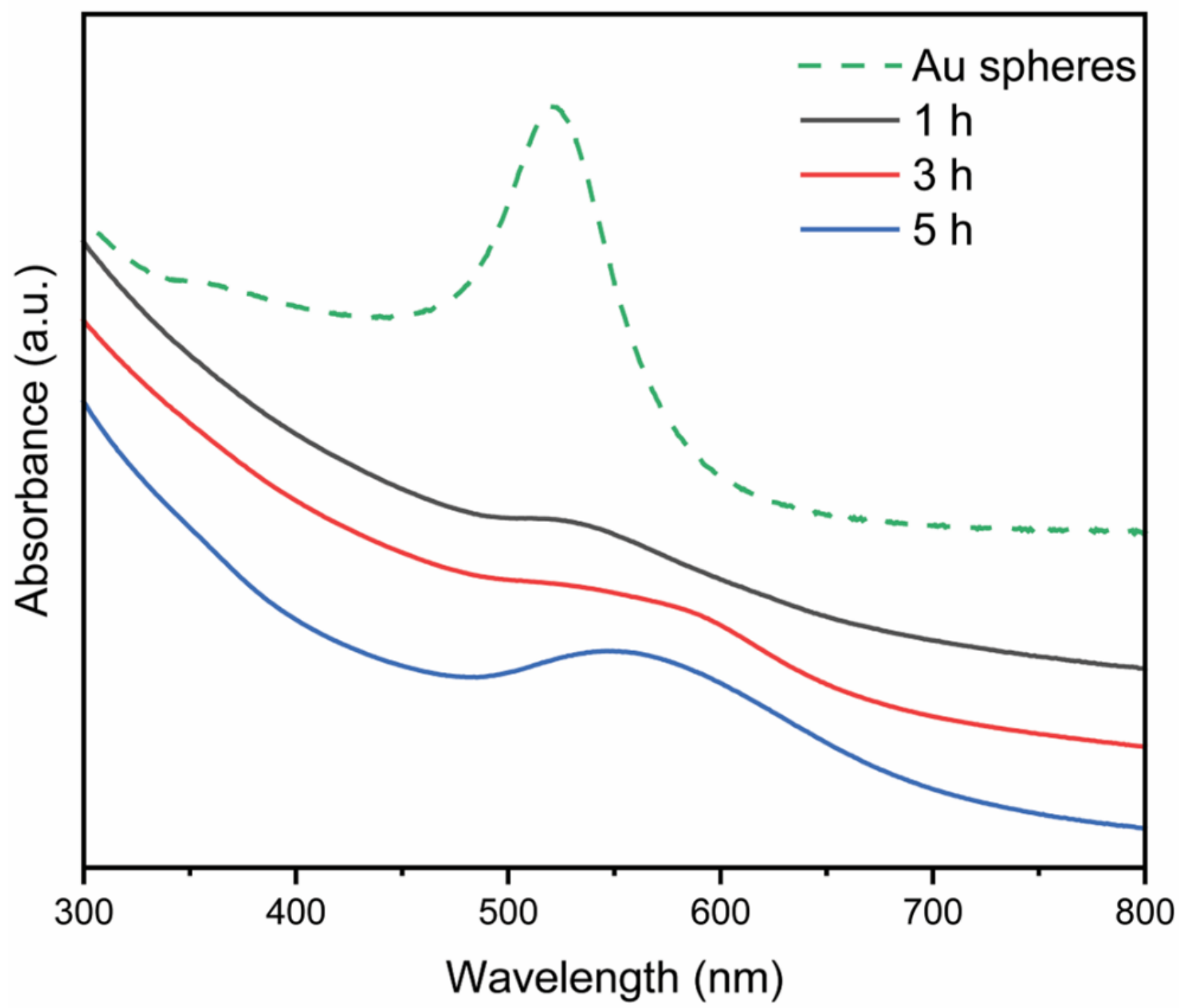

Fig. S7. Time-elapsed UV-vis spectra of the bimetallic nanocrystals synthesized when the total number of 10-nm Au spherical seeds was increased by 3-fold relative to the standard protocol. At *t* = 1 h (black trace), the LSPR peak red-shifted and damped when compared to that of the Au nanospheres (green trace). As the reaction time was increased to 3 h, the peak further red-shifted. To investigate this red shift and damping of the LSPR peak, we also recorded and compared the UV-vis spectra recorded from aqueous suspensions of the x4, x3, x1, and x1 samples obtained at *t* = 5 h (fig. S8). The suspensions remained stable during the UV-vis measurements, with no observable aggregation or sedimentation.

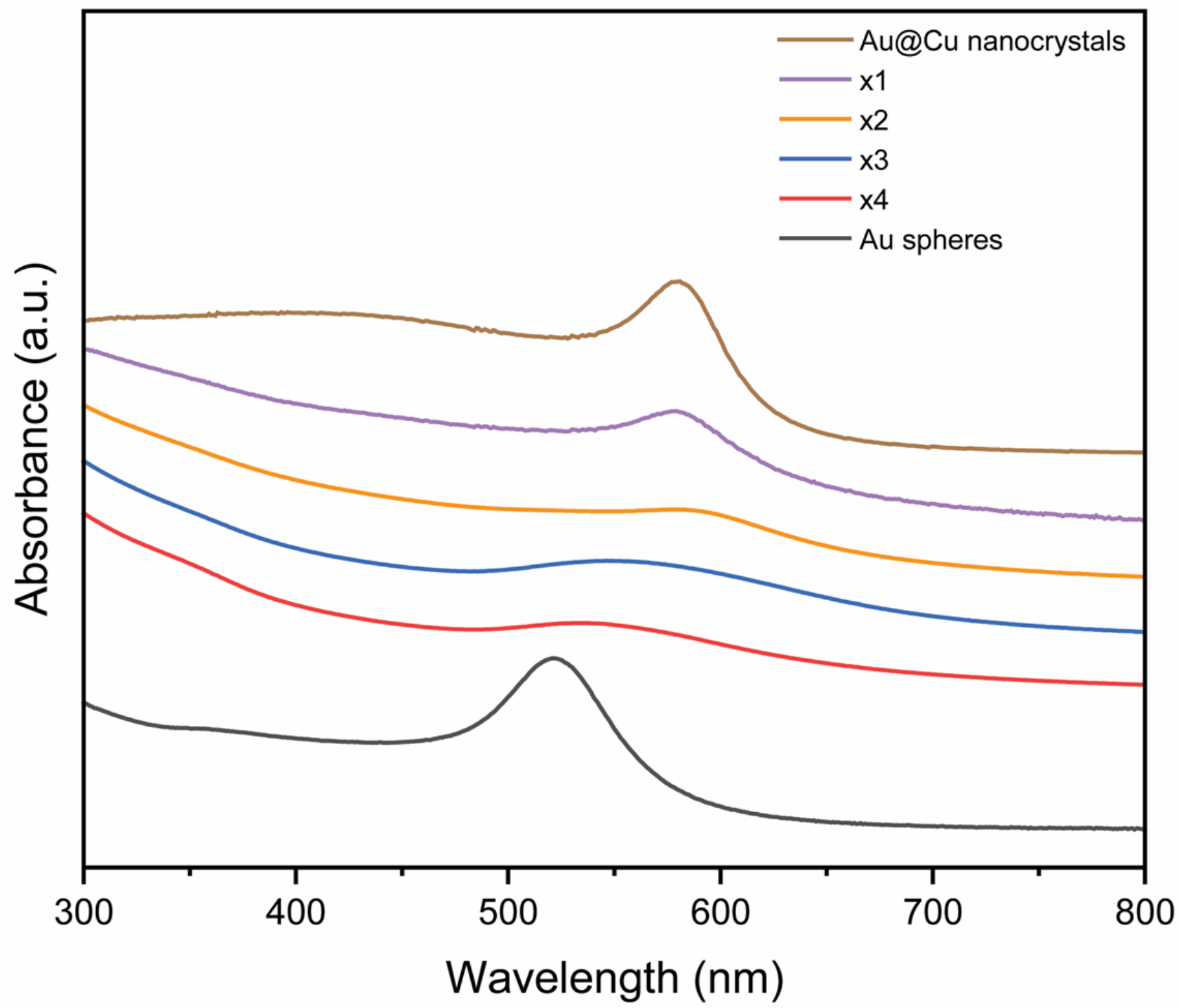

Fig. S8. UV-vis spectra recorded from aqueous suspensions of the 10-nm Au spheres, Au-Cu bimetallic nanocrystals synthesized by adding different numbers of the spherical seeds (t = 5 h), and Au@Cu core-shell nanocrystals. The Au nanospheres and Au@Cu nanocrystals with a symmetric and relatively thick Cu shell (fig. S9) served as references of pure Au and pure Cu. For the x4 sample, the LSPR peak featured a red shift, together with significant damping (red trace), when compared to the Au nanospheres. As the volume of the Cu component on each particle was increased from the x4 to the x2 sample, the LSPR peak progressively red-shifted to 580 nm. The changes could be attributed to the Au-Cu alloying that altered the electronic structure and plasmonic property of the nanocrystals. However, the peak stopped shifting with further enlargement of the Cu portion in the case of the x1 sample and Au@Cu reference, while it began to intensify. The increase in peak intensity indicated that pure Cu started to dominate the LSPR peak over pure Au and Au-Cu bimetallic in the samples.

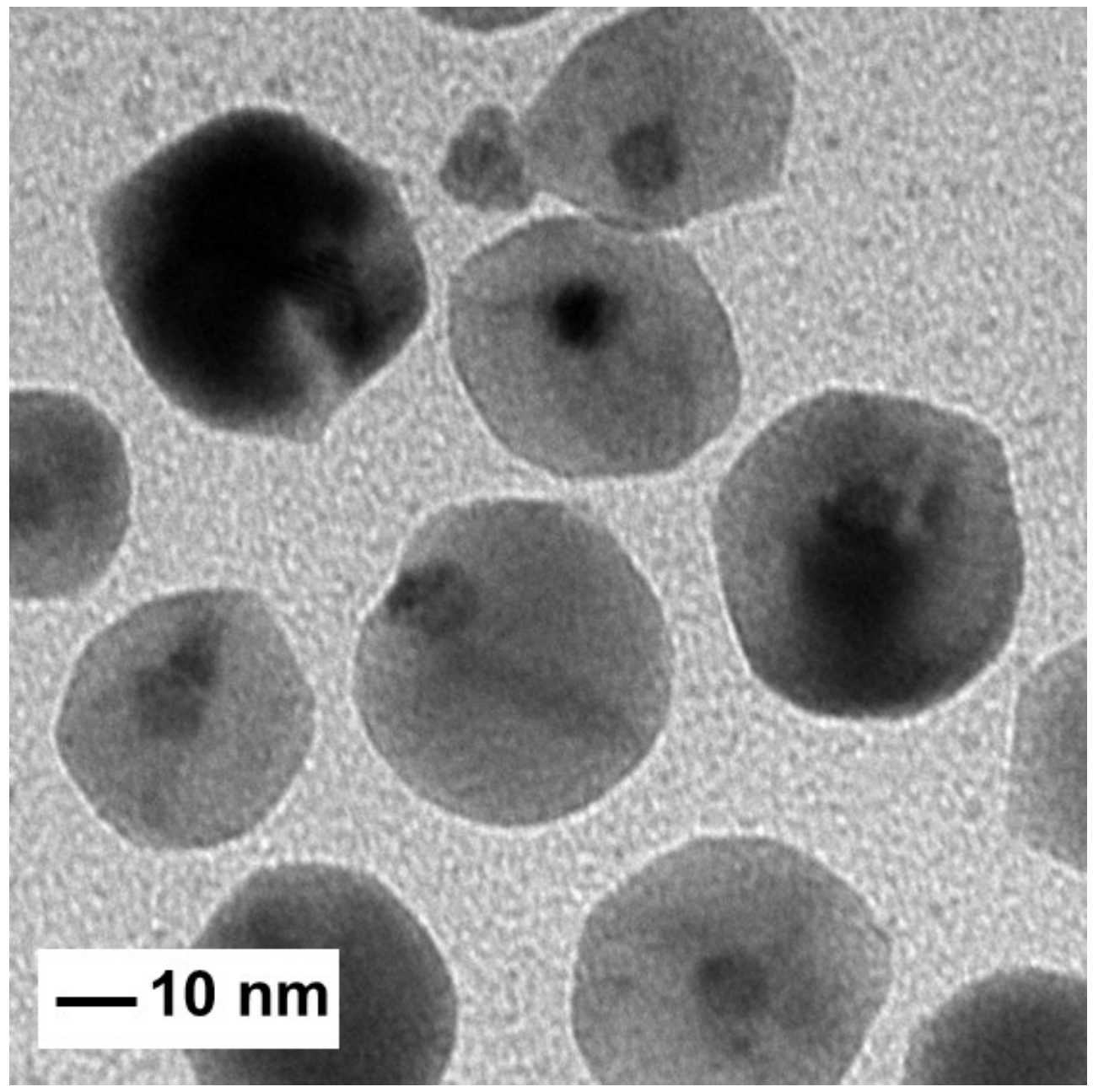

Fig. S9. TEM image of the Au@Cu core-shell nanocrystals prepared by adding 3.11×10^{10} of the Au spherical seeds (the standard protocol) while increasing the amount of Cu(II) precursor from 3.08 μmol to 61.6 μmol.

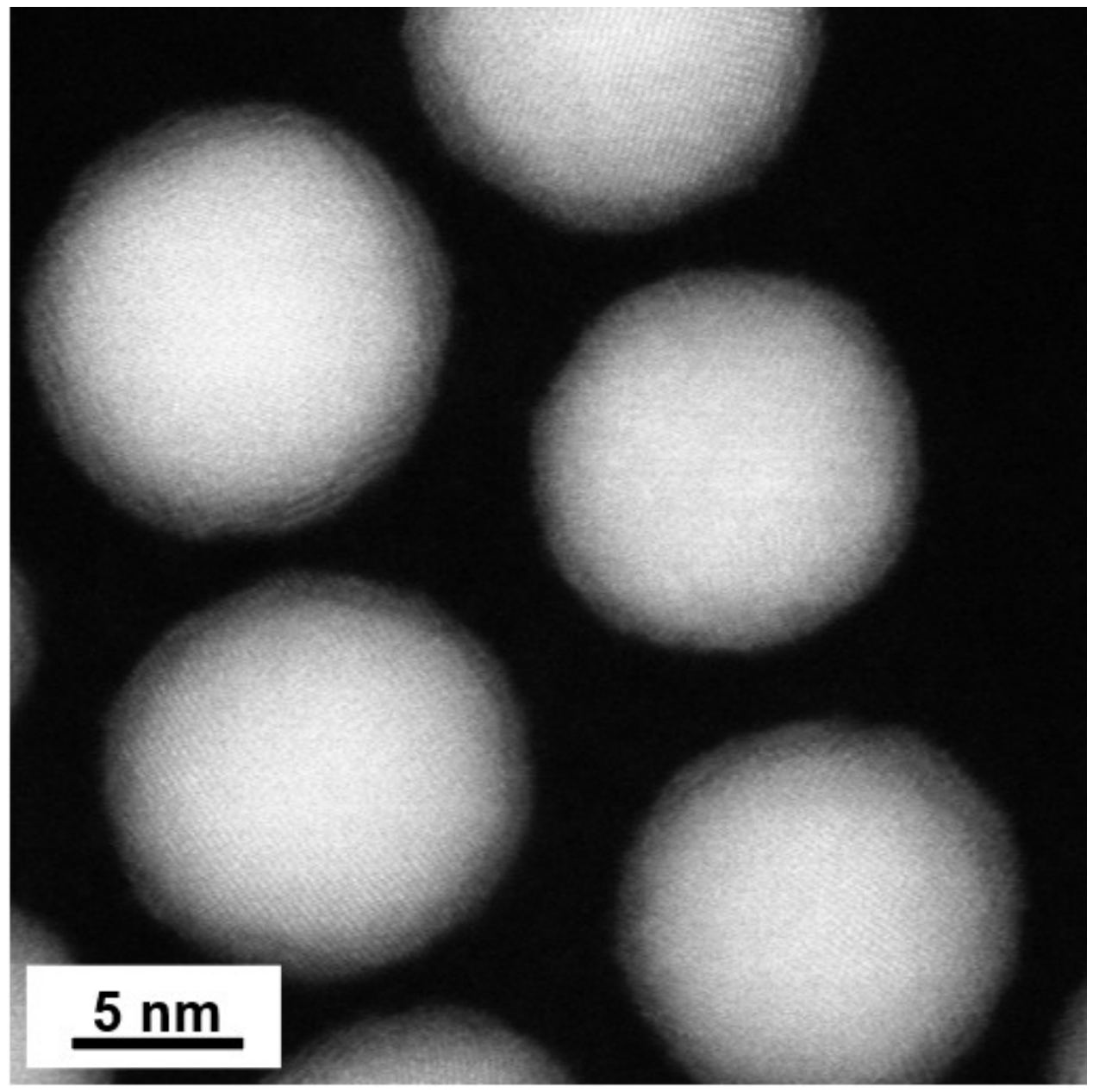

Fig. S10. STEM image of the x3 sample harvested at t = 1 h into the synthesis.

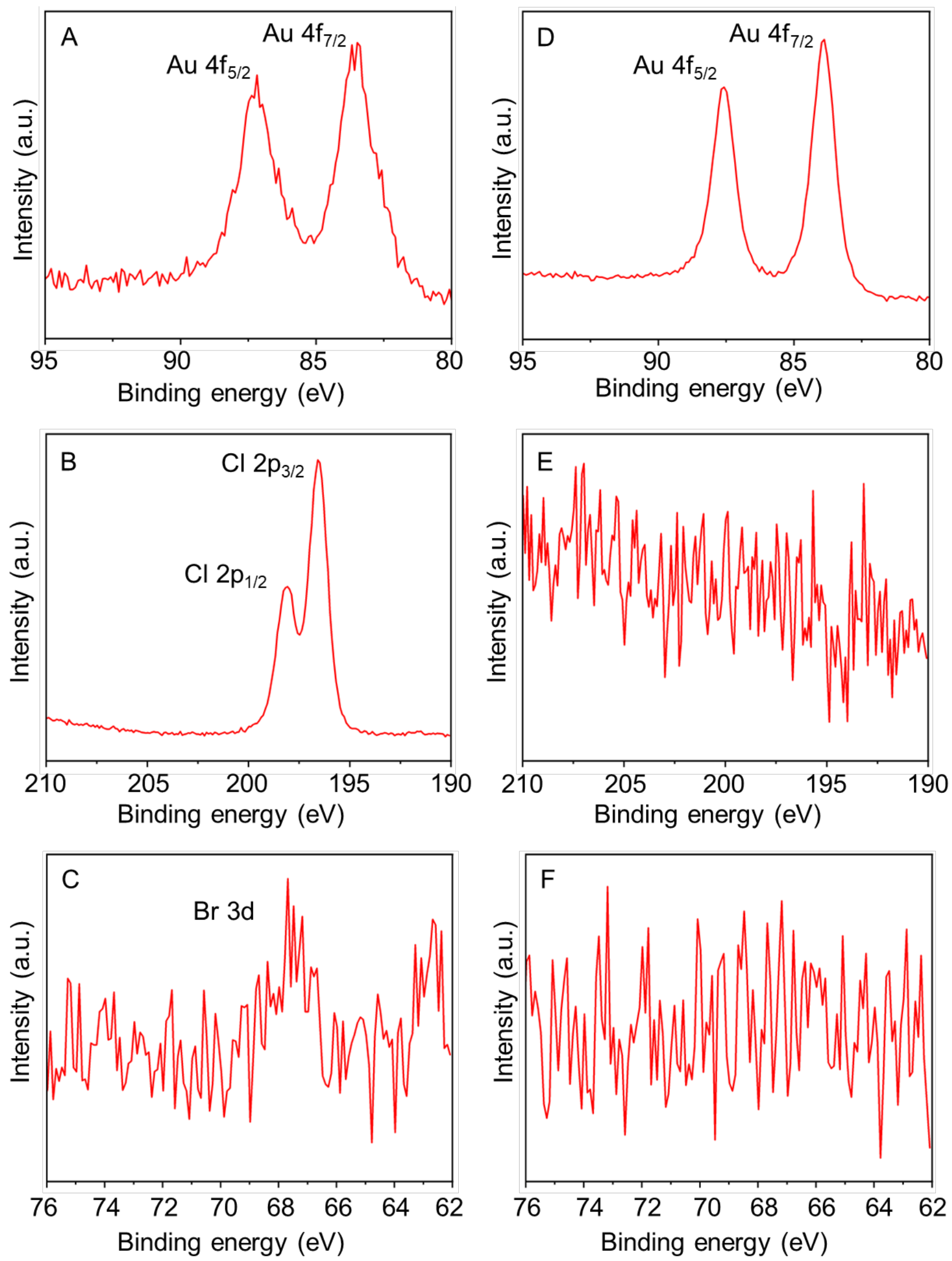

Fig. S11. X-ray photoelectron spectroscopy (XPS) spectra of Au 4f, Cl 2p, and Br 3d for the (A-C) 10-nm Au spherical seeds and (D-F) x3 sample. As expected, prominent Au doublet peaks were observed in the Au 4f spectra of both samples. For the Au nanospheres, strong doublet peaks were observed in the Cl 2p region, together with a small peak in the Br 3d region. The presence of halide peaks indicated that the particles were capped by cetyltrimethylammonium bromide/chloride (CTAB/C). In contrast, no Cl nor Br peaks were observed in the Cl 2p and Br 3d spectra of the x3 sample after the deposition of Cu, suggesting that hexadecylamine (HDA) instead of CTAB/C actually bound to the surface of the nanocrystals.

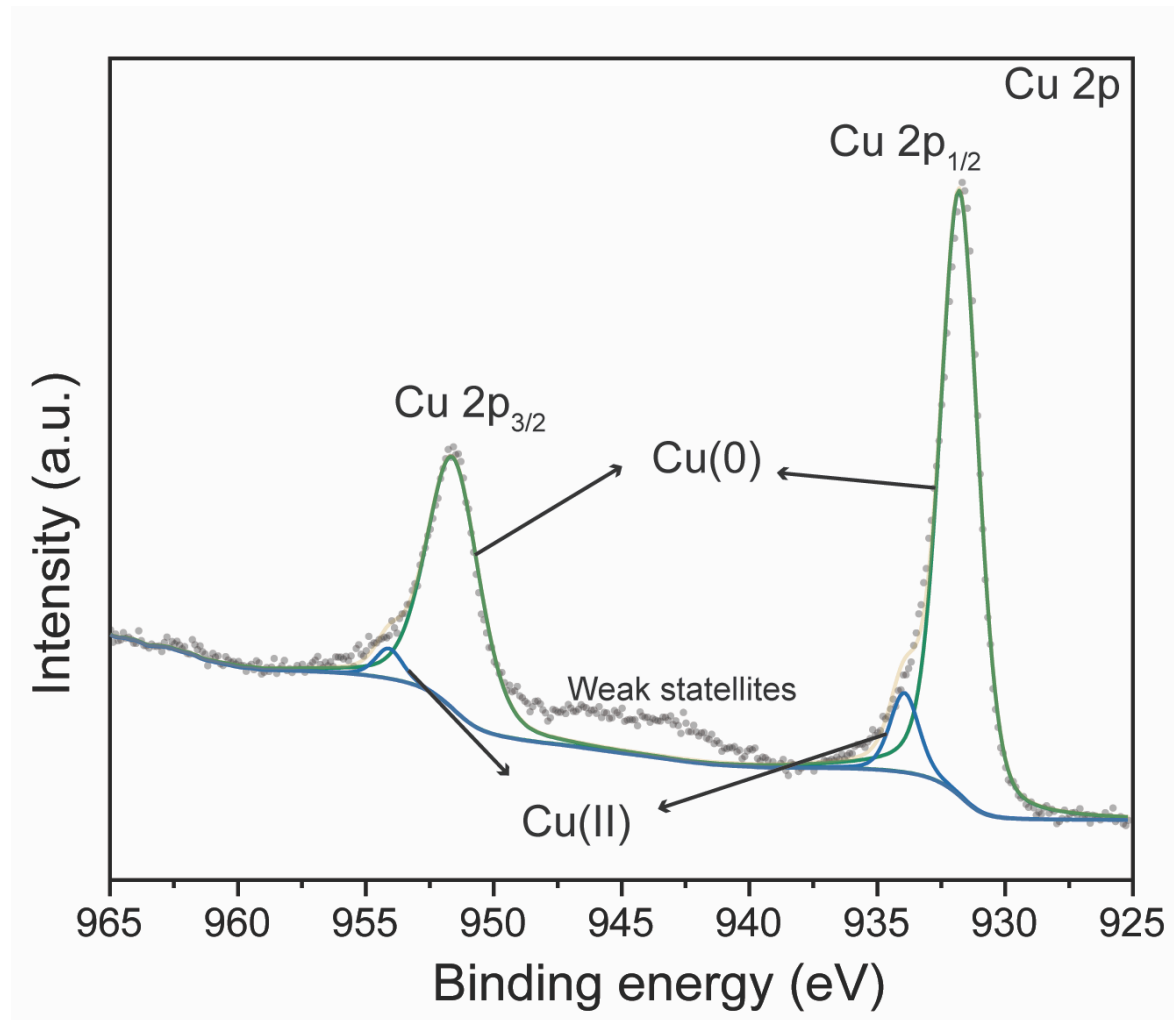

Fig. S12. XPS spectra of Cu 2p for the x3 sample.

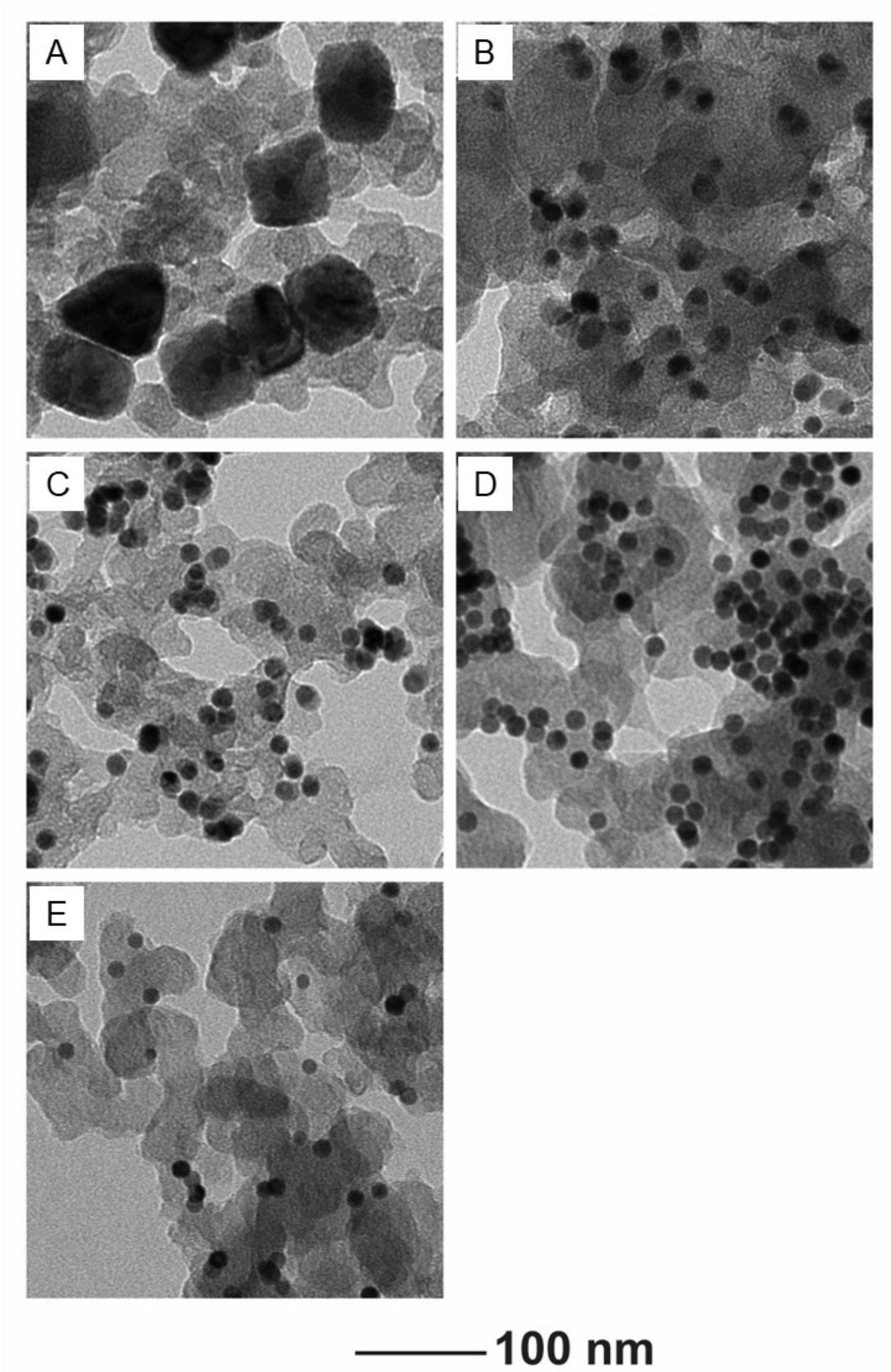

Fig. S13. TEM images of the catalysts supported on carbon black. The catalysts are based on the (A) Au@Cu, (B) x1 sample, (C) x3 sample, (D) x3 (1 h) sample, and (E) Au spheres, respectively. The scale bar applies to all panels.

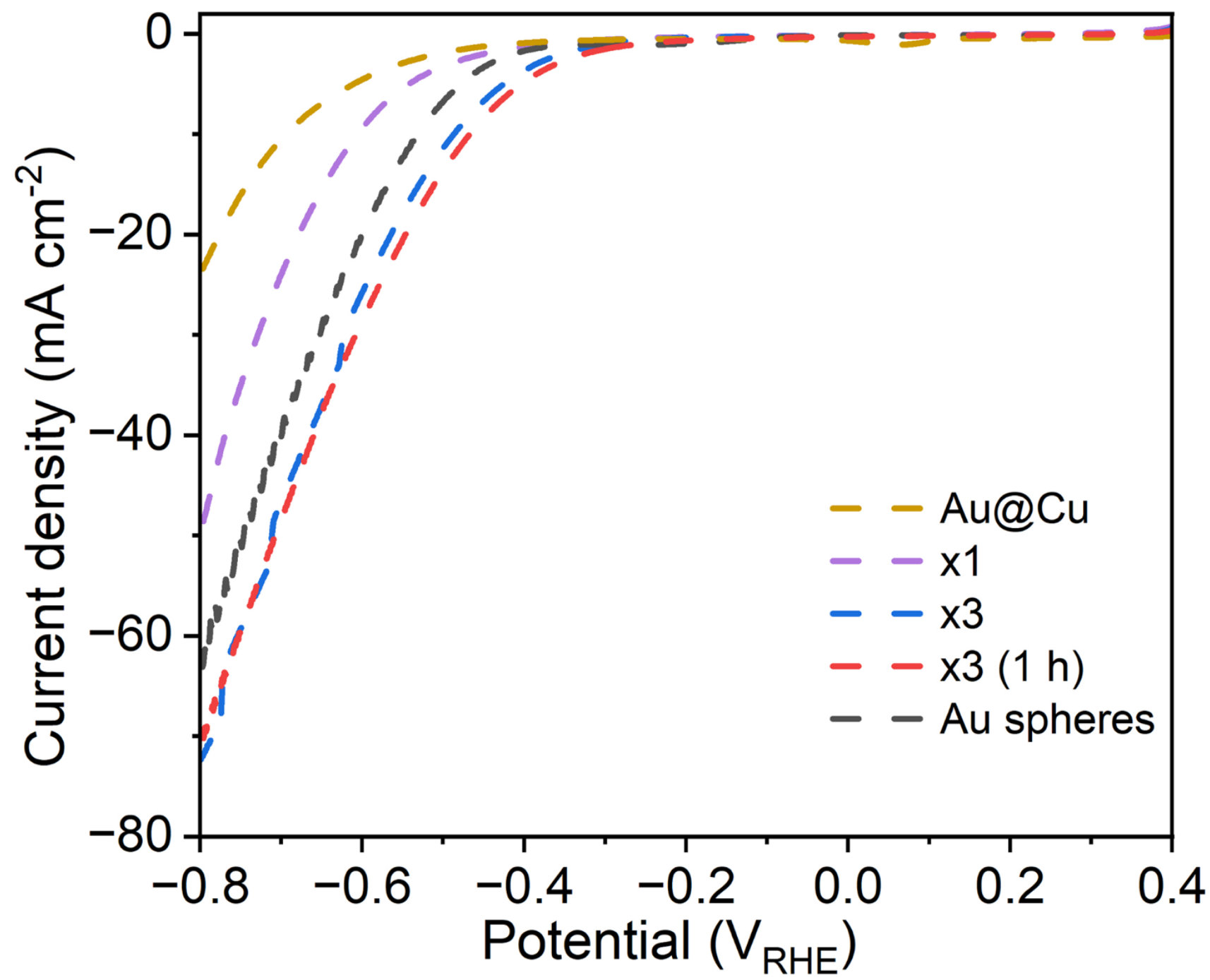

Fig. S14. Linear sweep voltammetry (LSV) curves of the catalysts based on Au@Cu, x1, x3, x3 (1 h), and Au spheres, respectively, in 1 M aqueous KOH.

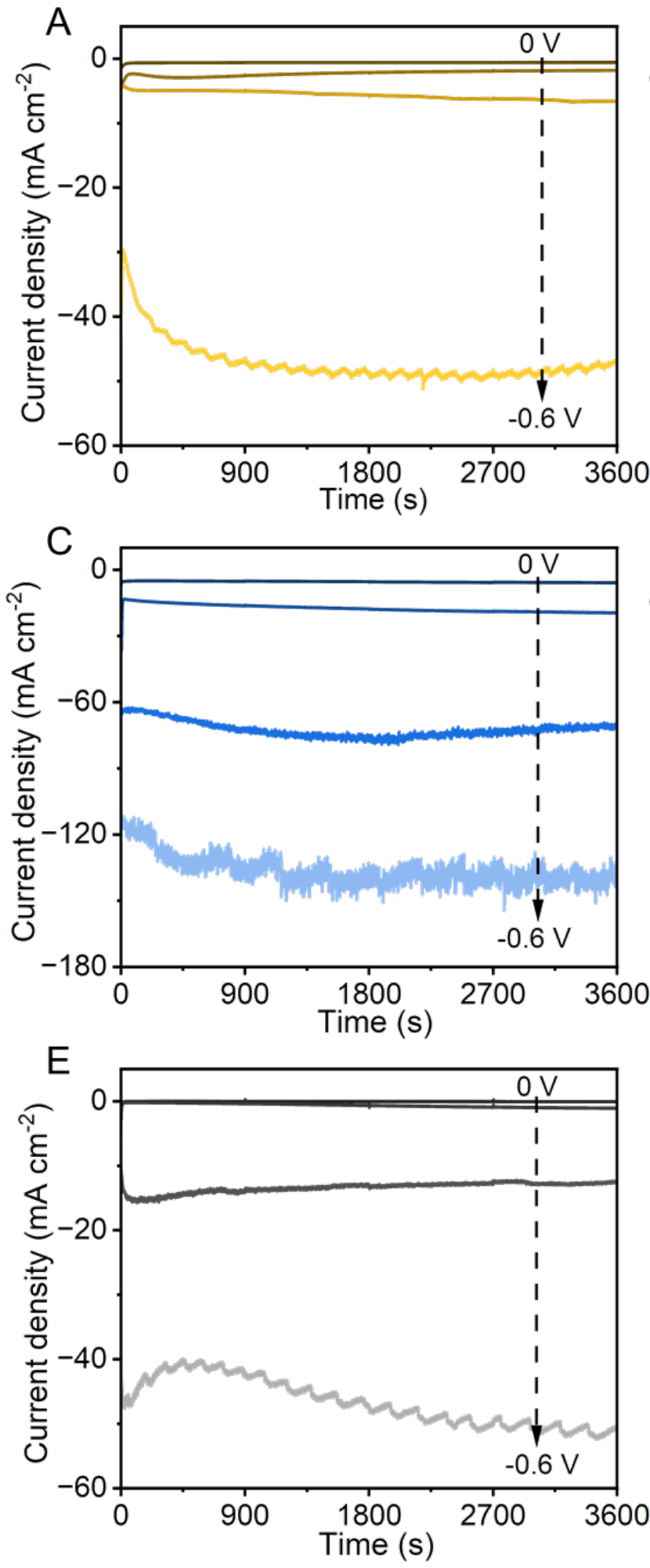

Fig. S15. Time-dependent current density curves of the catalysts based on (A) Au@Cu, (B) x1, (C) x3, (D) x3 (1 h), and (E) Au spheres, respectively.

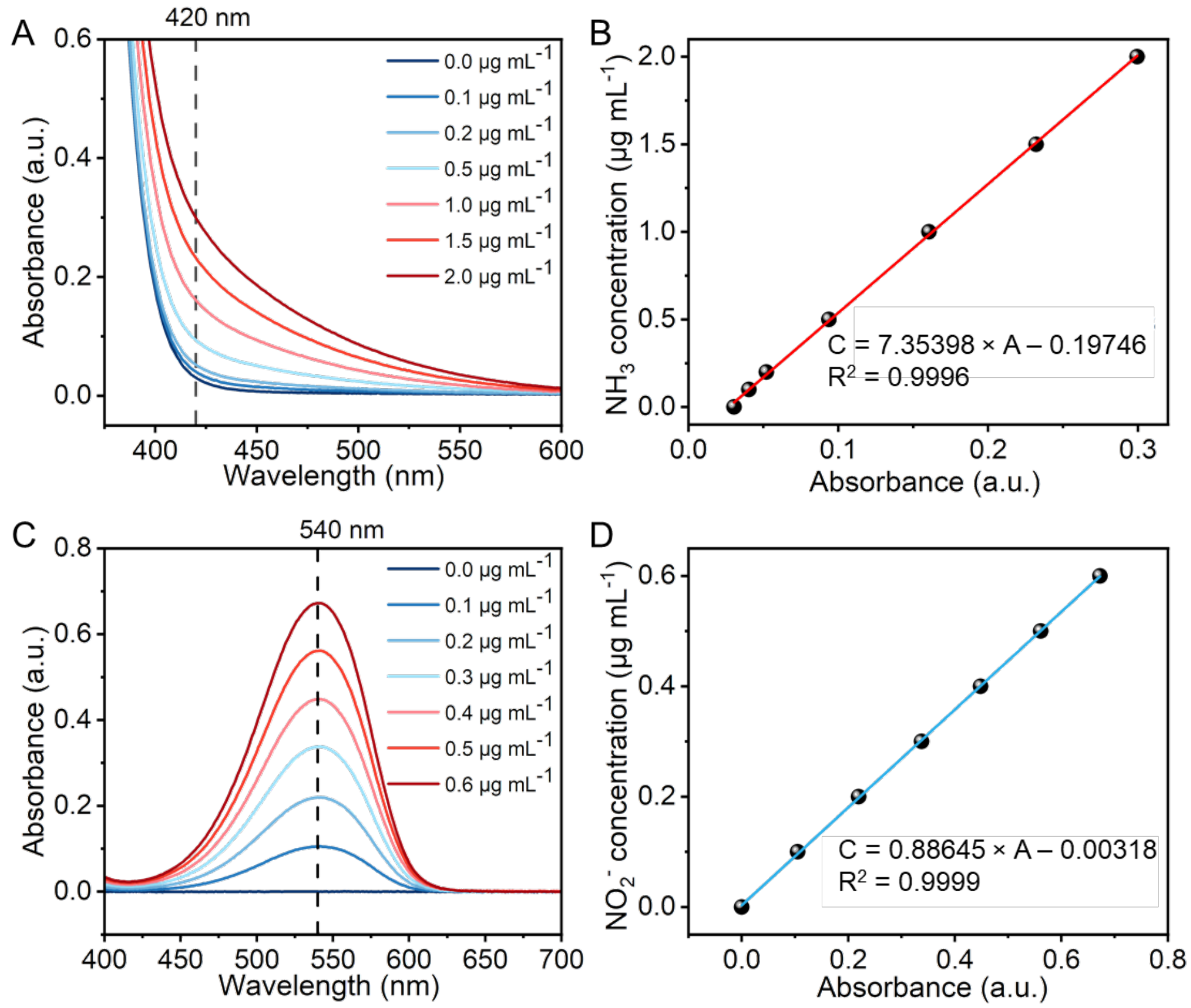

Fig. S16. Spectra of calibration solutions used to determine the ammonia and nitrite concentrations: (A) UV-vis spectra (375-600 nm) of one set of stock solutions, (B) linear fitting for the absorbance at 420 nm, (C) UV-vis spectra (400-700 nm) of another set of stock solutions, and (D) linear fitting for the absorbance at 540 nm.

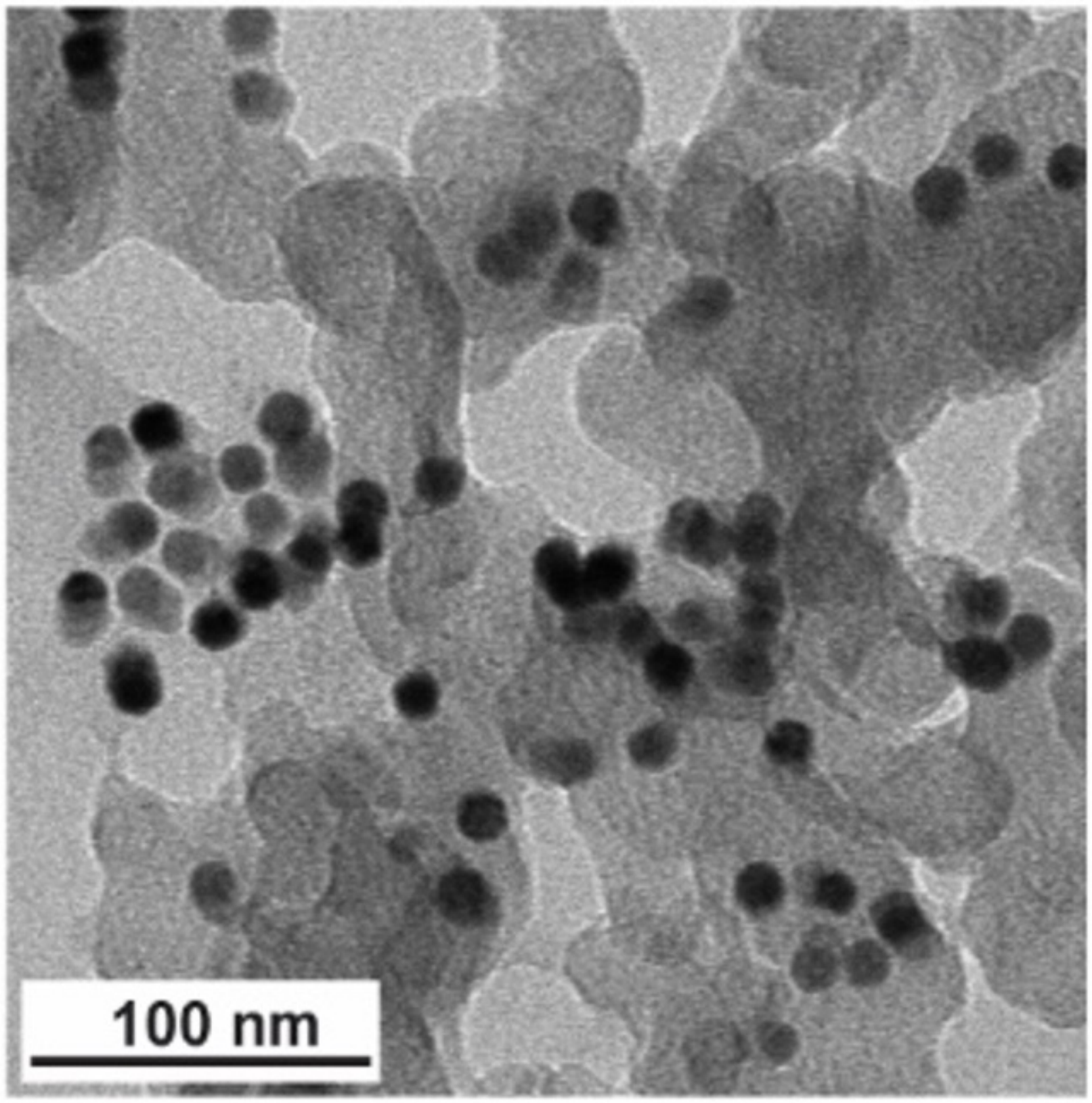

Fig. S17. TEM image of the catalyst based on the x3 sample after the stability test.

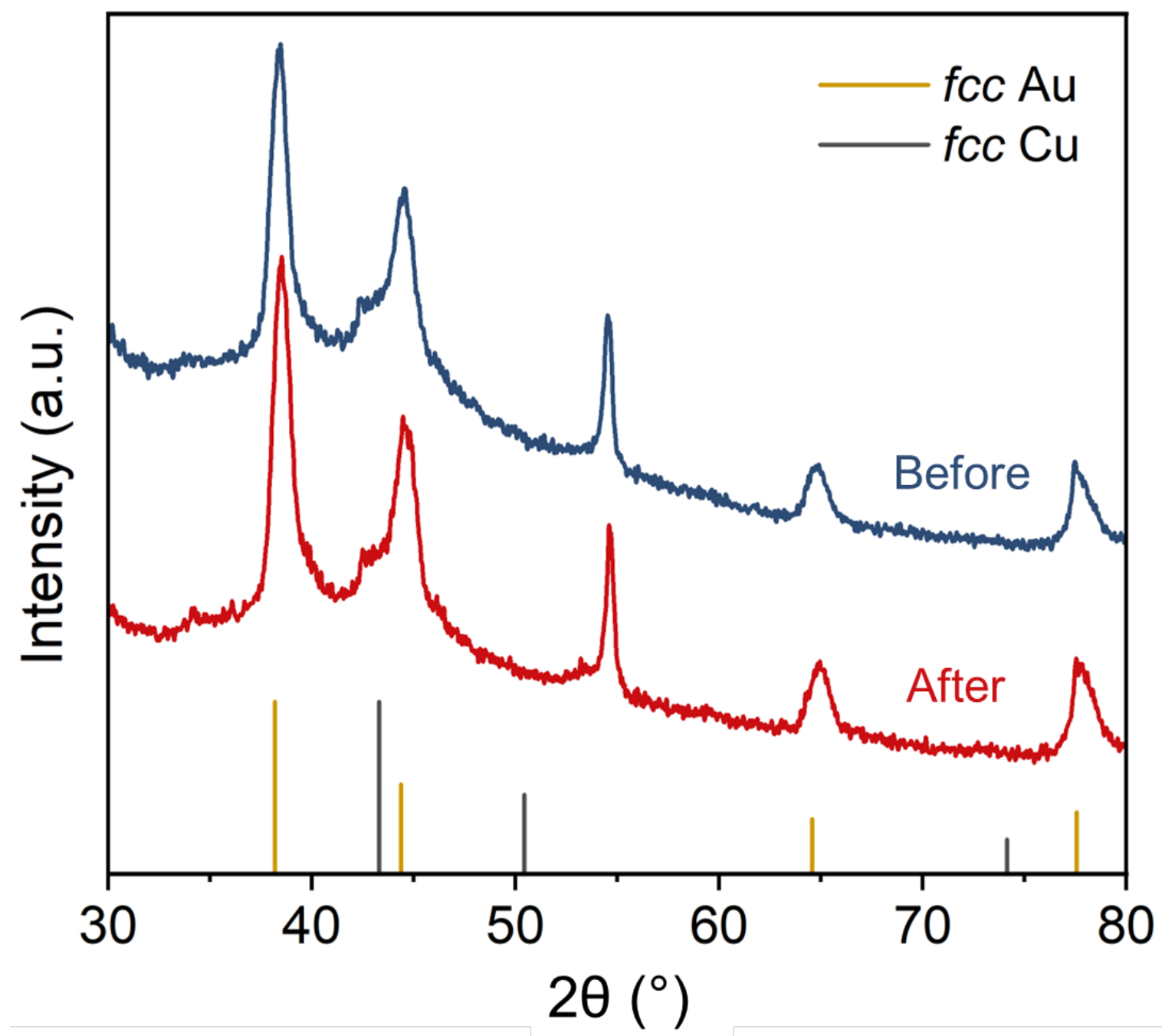

Fig. S18. X-ray diffraction (XRD) patterns of the catalyst based on the x3 sample before (blue) and after (red) the stability test.

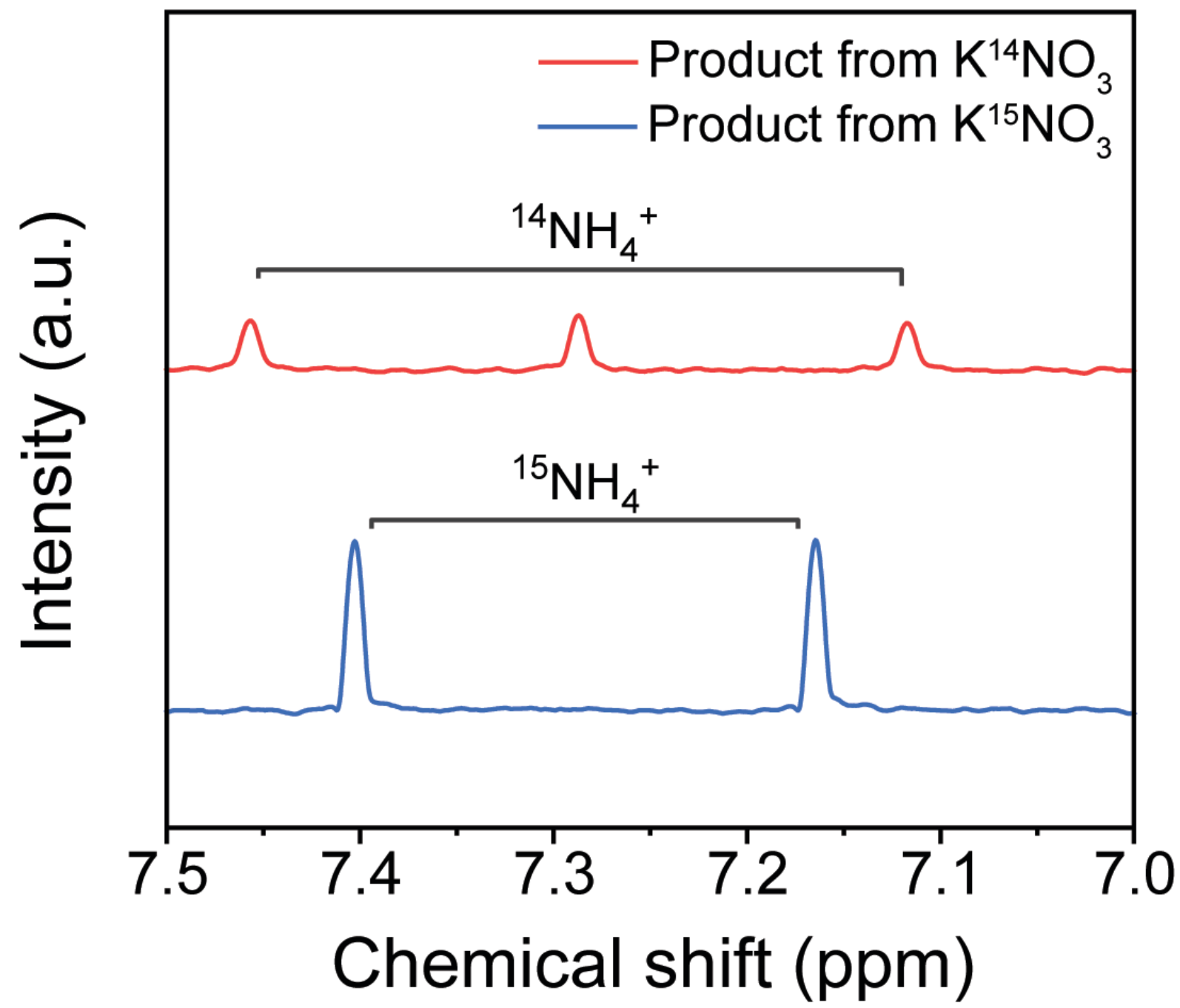

Fig. S19. Proton nuclear magnetic resonance (^1H NMR) spectra of different electrolytes after nitrate reduction reaction (NO_3RR) with the x3 sample (at –0.2 V_{RHE}), using $^{14}NO_3^-$ or $^{15}NO_3^-$ as the nitrogen source.

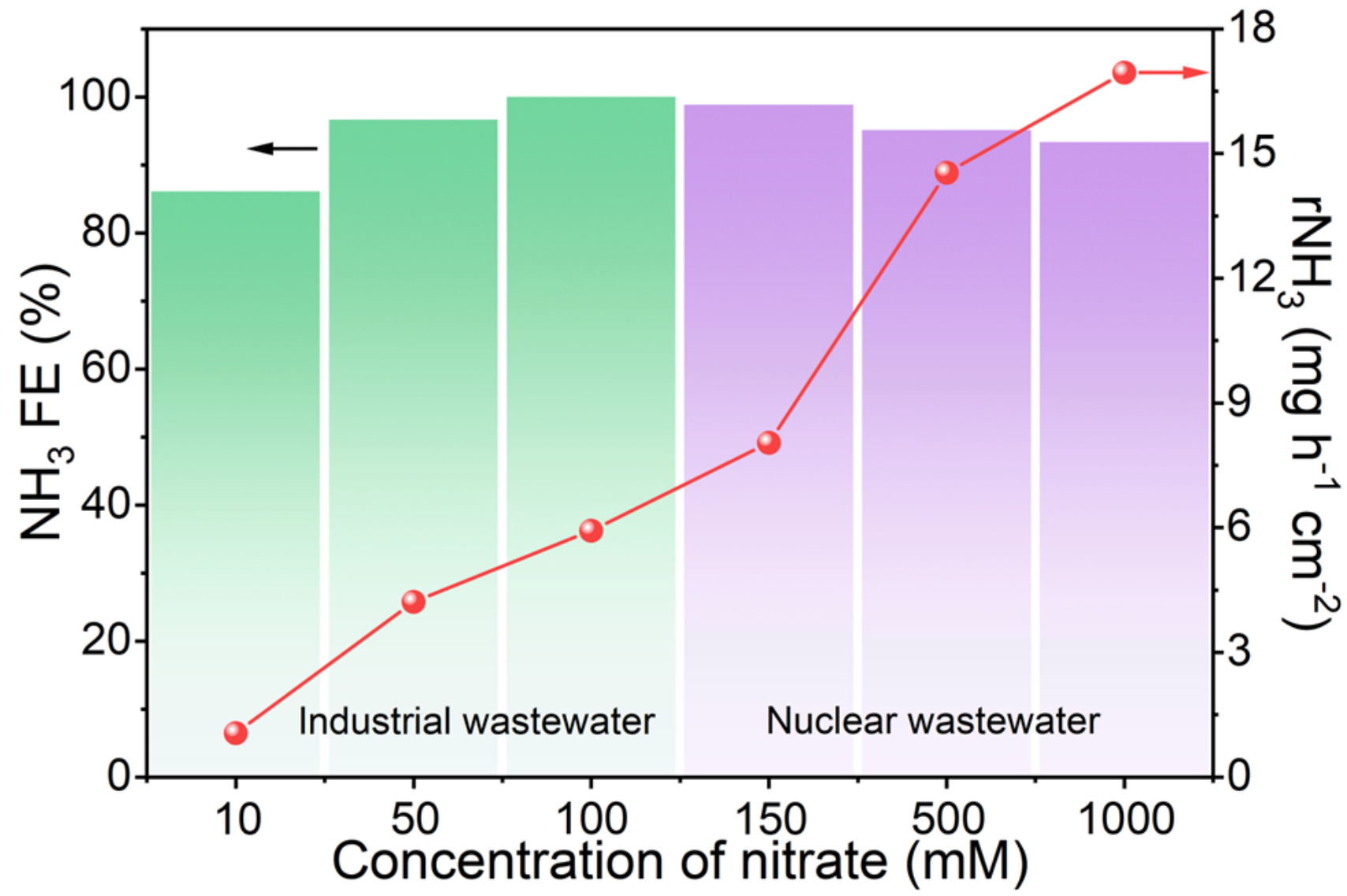

Fig. S20. The NH_3 Faradaic efficiency (FE) values and yield rates of the catalyst based on the x3 sample at –0.4 V_{RHE} in 1 M KOH at different concentrations of KNO_3.

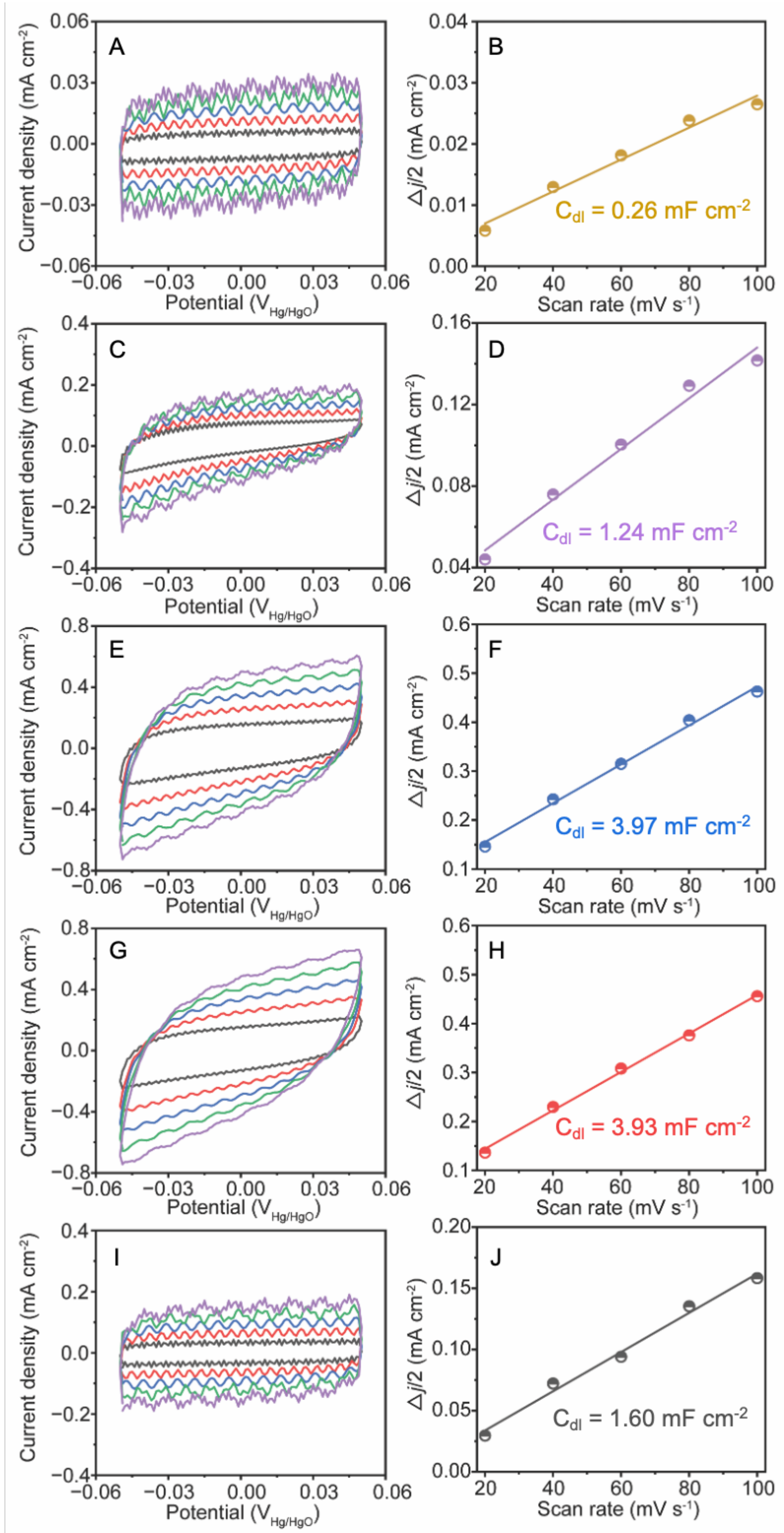

Fig. S21. Cyclic voltammetry (CV) curves and the linear fitting between current density and scan rate for catalysts based on (A, B) Au@Cu, (C, D) x1, (E, F) x3, (G, H) x3 (1 h), and (I, J) Au spheres. The alloying between the deposited Cu and the underlying Au increased electrochemically

active surface area (ECSA), contributing to the increase in overall current density. The ECSA decreased dramatically at excessive Cu deposition (the x1 sample) due to the increase in particle size and the formation of a Cu-dominant surface (fig. S26). When the Au spheres were fully encapsulated by Cu, the ECSA reached a minimum.

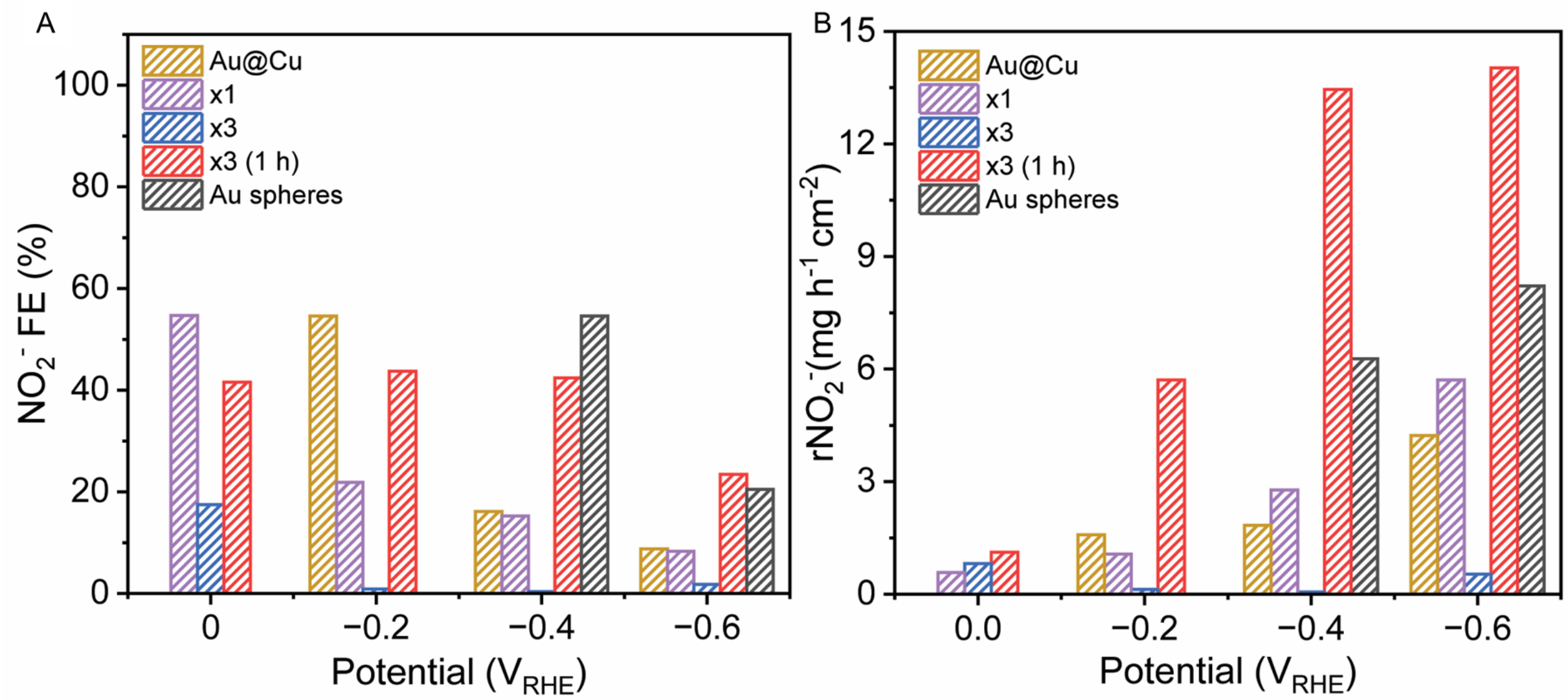

Fig. S22. Comparison of the (A) NO_2^- FE values and (B) NO_2^- yield rates obtained for different catalysts. The FE for NO_2^- decreased as the potential was reduced, with the x3 sample showing the most dramatic decrease and being stabilized at a low level of less than 2%. The x3 (1 h) catalyst exhibits the highest NO_2^- production rate across all applied potentials, even when its FE is not the highest.

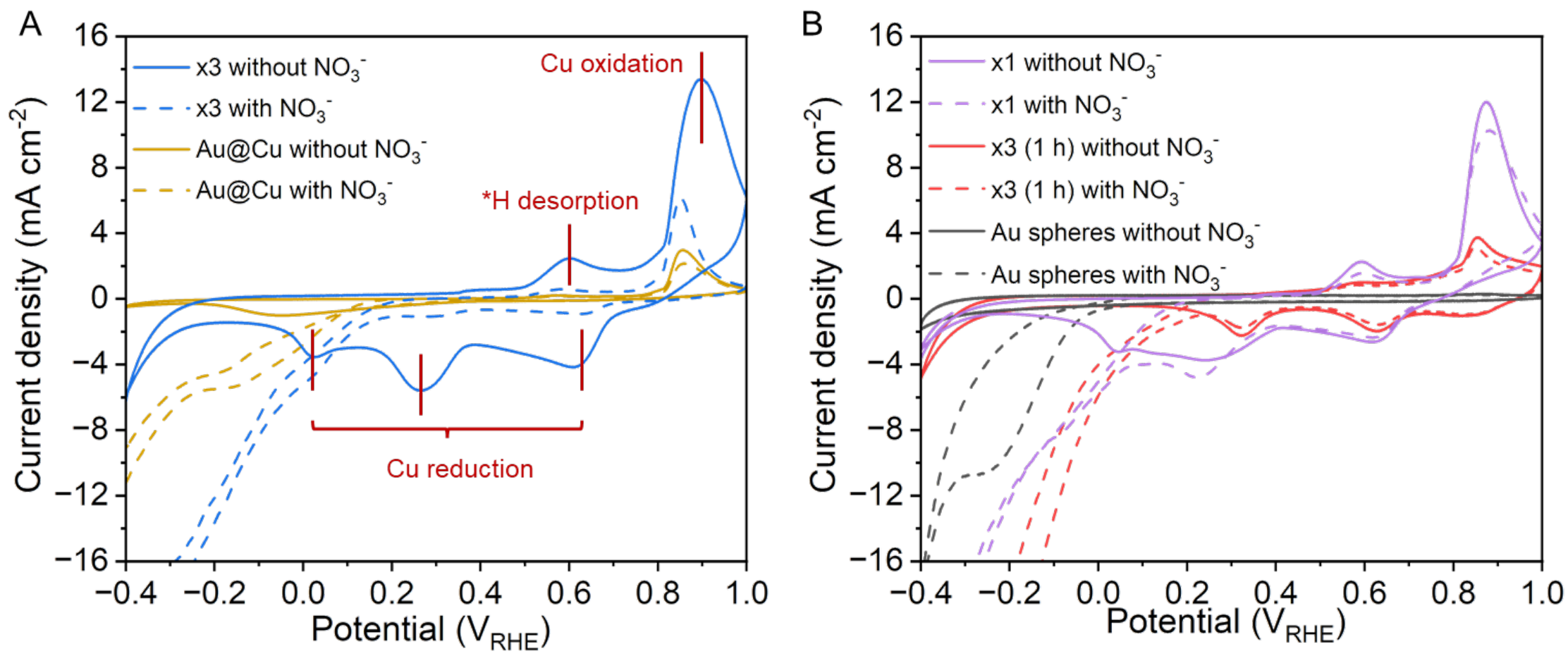

Fig. S23. CV curves of *H peaks measured in 1 M KOH with and without NO_3^- over the (A) x3, Au@Cu samples and (B) x1, x3 (1 h), and Au sphere samples.

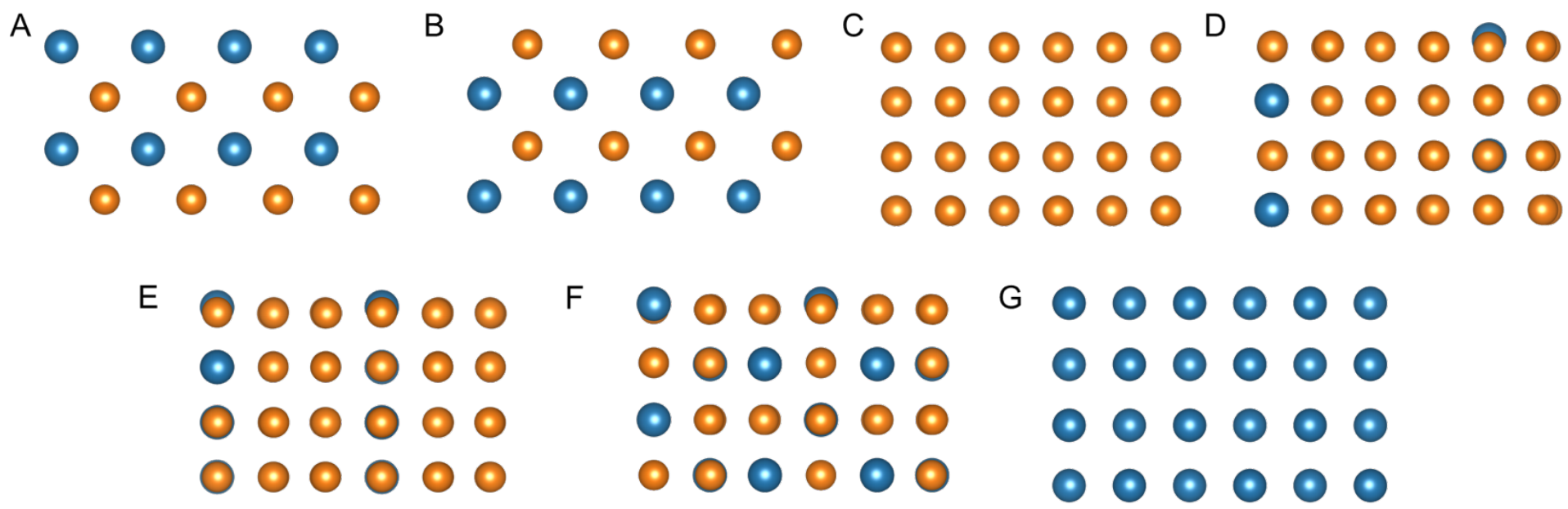

Fig. S24. Simplified models for DFT simulations of (A) *fct*-Au, (B) *fct*-Cu, (C) pure Cu, (D) Au_2Cu_{34}, (E) Au_4Cu_{32}, (F) Au_8Cu_{28}, and (G) pure Au structures (Cu atom in orange and Au atom in blue).

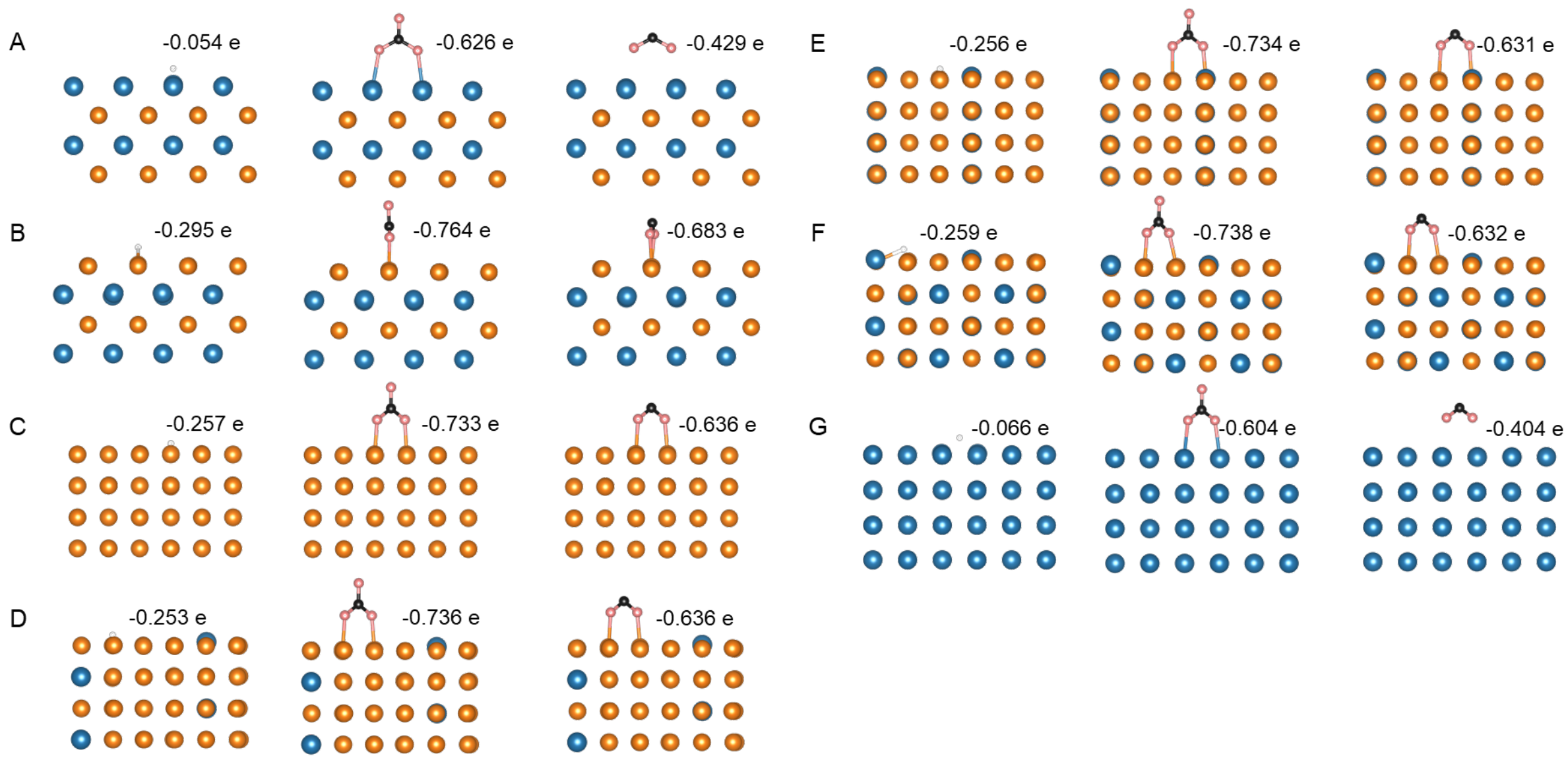

Fig. S25. Adsorption configurations and Bader charge of *H, $*NO_3$, and $*NO_2$ on (A) *fct*-Au, (B) *fct*-Cu, (C) pure Cu, (D) Au_2Cu_{34}, (E) Au_4Cu_{32}, (F) Au_8Cu_{28}, and (G) pure Au, respectively (orange for Cu atom, blue for Au atom, white for H atom, pink for O atom, and black for N atom).

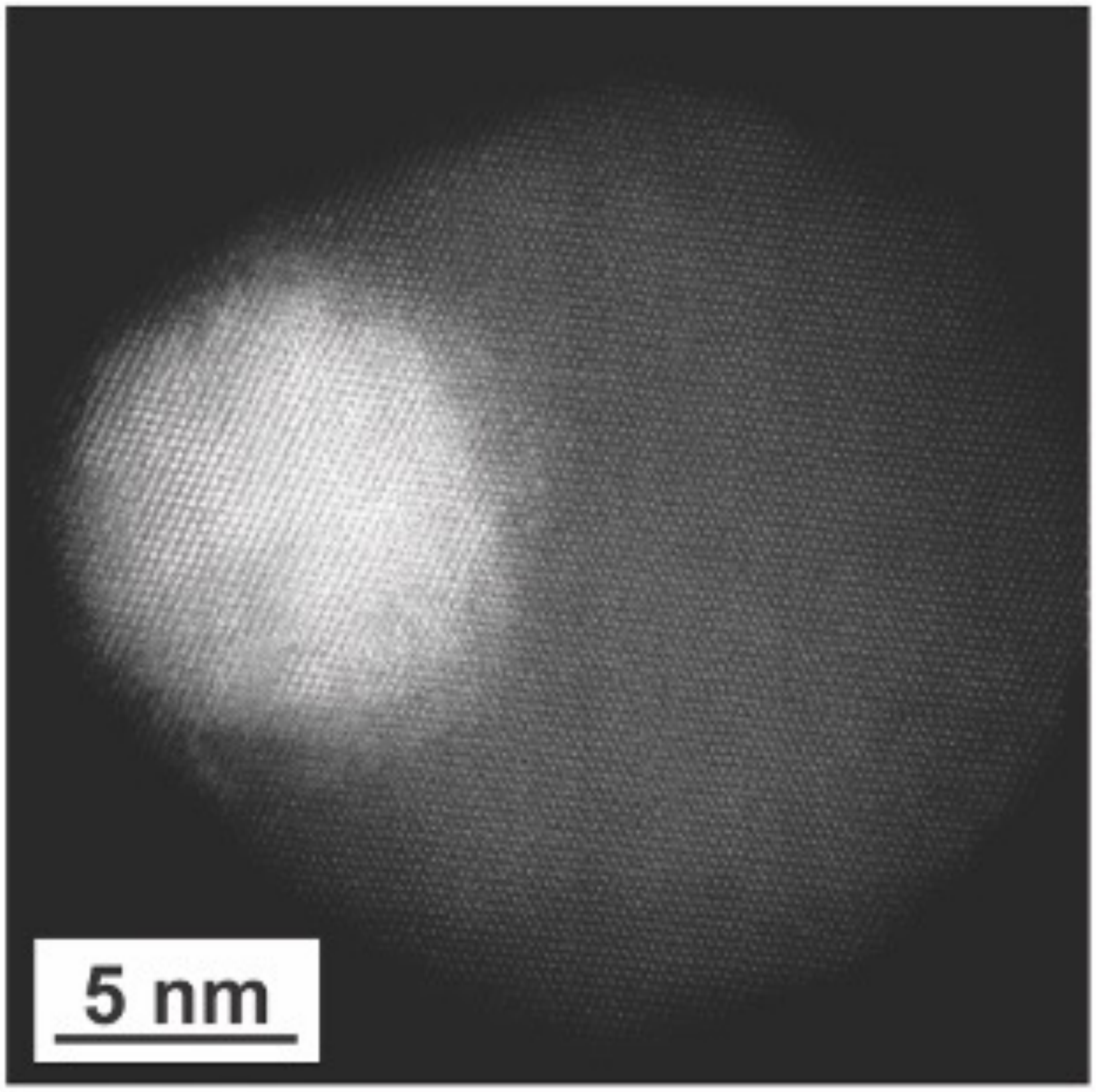

Fig. S26. HAADF-STEM image of an individual particle from the x1 sample.

Table S1. Concentrations of the aqueous suspension of Au spherical seeds used for the synthesis of the Au-Cu bimetallic nanocrystals.

**Sample**	**x1 (standard)**	**x2**	**x3**	**x4**
**Concentration of the Au seeds (particle/mL)**	1.56×10^{11}	3.12×10^{11}	4.68×10^{11}	6.20×10^{11}

Table S2. Comparison of the electrochemical NO_3RR performance of the catalyst based on the x3 sample with many other Cu-based electrocatalysts reported in the literature.

Catalyst	Electrolyte	Potential (V_{RHE})	NH_3 FE	NH_3 yield rate (mg h^{-1} cm^{-2})	Ref.
**x3**	**1 M KOH + 0.1 M KNO_3**	**–0.20~–0.60**	**>99.0%**	**10.80 (at –0.6 V_{RHE})**	**This work**
**x3**	**1 M KOH + 1 M KNO_3**	**–0.40**	**93.4%**	**16.90**	**This work**
Cu/CuAu ordered SAA NCs	1 M KOH + 1 M KNO_3	–0.50	85.5%	28.80 (at –0.6 V_{RHE})	(*34*)
CuPd nanocubes	1 M KOH + 1 M KNO_3	–0.50	92.5%	21.20 (at –0.6 V_{RHE})	(*63*)
Cu/Cu_2O	200 ppm NO_3^--N + 0.5 M Na_2SO_4	–0.85	95.8%	4.20	(*74*)
Cu-NBs-100	1 M KOH + 0.1 M KNO_3	–0.15	95.3%	11.10	(*75*)
Ru_1Cu_{10}/GO	1 M KOH + 0.1 M KNO_3	–0.050	98.0%	6.46	(*76*)
Cu-Fe_3O_4	0.1 M KOH + 0.1 M KNO_3	–0.60	99.8%	7.20	(*77*)
PdCuRu MS	0.1 M KOH + 0.01 M KNO_3	–0.20	95.0%	0.85	(*57*)
AuCu NDs	1 M KOH + 0.075 M KNO_3	–0.70	93.3%	16.80 (at –0.8 V_{RHE})	(*54*)
Cu_2O/Cu@mesoPdCu	1 M KOH + 0.1 M KNO_3	0.10	96.2%	1.33	(*78*)
meso-*i*-$AuCu_3$@*ultra*-Au	1 M KOH + 1 M KNO_3	–0.55	98.1%	20.60	(*55*)
PdCu MCs	0.1 M KOH + 0.01 M KNO_3	–0.20	96.6%	0.56	(*56*)

Table S3. ECSAs of the catalysts based on the Au@Cu, x1, x3, x3 (1 h), and Au spheres.

Samples	Au@Cu	x1	x3	x3 (1 h)	Au spheres
ECSA	6.5	31.1	99.4	98.2	40.1

REFERENCES

1. K. D. Gilroy, A. Ruditskiy, H.-C. Peng, D. Qin, Y. Xia, Bimetallic nanocrystals: Syntheses, properties, and applications. *Chem. Rev.* **116**, 10414–10472 (2016).

2. L. Shi, J. Song, Y. Yang, L. Yang, Z. Dai, L. Yao, W. Jiang, Advances in the design strategy of bimetallic catalysts for the electrocatalytic reduction of CO_2. *J. Mater. Chem. A* **13**, 2478–2504 (2025).

3. A. N. Kuhn, H. Zhao, U. O. Nwabara, X. Lu, M. Liu, Y.-T. Pan, W. Zhu, P. J. A. Kenis, H. Yang, Engineering silver-enriched copper core-shell electrocatalysts to enhance the production of ethylene and C_{2+} chemicals from carbon dioxide at low cell potentials. *Adv. Funct. Mater.* **31**, 2101668 (2021).

4. Y. E. Jeon, Y. N. Ko, J. Kim, H. Choi, W. Lee, Y. E. Kim, D. Lee, H. Y. Kim, K. T. Park, Selective production of ethylene from CO_2 over CuAg tandem electrocatalysts. *J. Ind. Eng. Chem.* **116**, 191–198 (2022).

5. L. V. Brown, H. Sobhani, J. B. Lassiter, P. Nordlander, N. J. Halas, Heterodimers: Plasmonic properties of mismatched nanoparticle pairs. *ACS Nano* **4**, 819–832 (2010).

6. S. Sheikholeslami, Y.-W. Jun, P. K. Jain, A. P. Alivisatos, Coupling of optical resonances in a compositionally asymmetric plasmonic nanoparticle dimer. *Nano Lett.* **10**, 2655–2660 (2010).

7. M. Mayer, L. Scarabelli, K. March, T. Altantzis, M. Tebbe, M. Kociak, S. Bals, F. J. García de Abajo, A. Fery, L. M. Liz-Marzán, Controlled living nanowire growth: Precise control over the morphology and optical properties of AgAuAg bimetallic nanowires. *Nano Lett.* **15**, 5427–5437 (2015).

8. J. Qiu, Q. N. Nguyen, Z. Lyu, Q. Wang, Y. Xia, Bimetallic Janus nanocrystals: Syntheses and applications. *Adv. Mater.* **34**, e2102591 (2022).

9. J.-H. Lee, G.-H. Kim, J.-M. Nam, Directional synthesis and assembly of bimetallic nanosnowmen with DNA. *J. Am. Chem. Soc.* **134**, 5456–5459 (2012).

10. J. Kim, J.-M. Kim, M. Ha, J.-W. Oh, J.-M. Nam, Polysorbate- and DNA-mediated synthesis and strong, stable, and tunable near-infrared photoluminescence of plasmonic long-body nanosnowmen. *ACS Nano* **15**, 19853–19863 (2021).

11. Z. Wu, L. Li, T. Liao, X. Chen, W. Jiang, W. Luo, J. Yang, Z. Sun, Janus nanoarchitectures: From structural design to catalytic applications. *Nano Today* **22**, 62–82 (2018).

12. Y. Ma, M. Shao, G. Wang, L. Guo, Y. Wang, F. Hao, F. Liu, X. Meng, C. Wang, Y. Xiong, Z. Fan, Controlled synthesis of metal-based Janus nanostructures for tandem electrocatalytic carbon dioxide reduction. *Sci. Bull.* **70**, 2330–2346 (2025).

13. B. Ni, P. He, W. Liao, S. Chen, L. Gu, Y. Gong, K. Wang, J. Zhuang, L. Song, G. Zhou, X. Wang, Surface oxidation of AuNi heterodimers to achieve high activities toward hydrogen/oxygen evolution and oxygen reduction reactions. *Small* **14**, 1703749 (2018).

14. Y. Ma, J. Yu, M. Sun, B. Chen, X. Zhou, C. Ye, Z. Guan, W. Guo, G. Wang, S. Lu, D. Xia, Y. Wang, Z. He, L. Zheng, Q. Yun, L. Wang, J. Zhou, P. Lu, J. Yin, Y. Zhao, Z. Luo, L. Zhai, L. Liao, Z. Zhu, R. Ye, Y. Chen, Y. Lu, S. Xi, B. Huang, C.-S. Lee, Z. Fan, Confined growth of silver–copper Janus nanostructures with {100} facets for highly selective tandem electrocatalytic carbon dioxide reduction. *Adv. Mater.* **34**, 2110607 (2022).

15. J. Huang, M. Mensi, E. Oveisi, V. Mantella, R. Buonsanti, Structural sensitivities in bimetallic catalysts for electrochemical CO_2 reduction revealed by Ag–Cu nanodimers. *J. Am. Chem. Soc.* **141**, 2490–2499 (2019).

16. M. B. Gawande, A. Goswami, F. X. Felpin, T. Asefa, X. Huang, R. Silva, X. Zou, R. Zboril, R. S. Varma, Cu and Cu-based nanoparticles: synthesis and applications in catalysis. *Chem. Rev.* **116**, 3722–3811 (2016).

17. D. Liu, L. Qiao, S. Peng, H. Bai, C. Liu, W. F. Ip, H. K. Lo, H. Liu, K. W. Ng, S. Wang, X. Yang, H. Pan, Recent advances in electrocatalysts for efficient nitrate reduction to ammonia. *Adv. Funct. Mater.* **33**, 2303480 (2023).

18. K. Zhang, Y. Liu, Z. Pan, Q. Xia, X. Huo, O. C. Esan, X. Zhang, L. An, Cu-based catalysts for electrocatalytic nitrate reduction to ammonia: Fundamentals and recent advances. *EES Catal.* **2**, 727–752 (2024).

19. Y.-Y. Lou, Q.-Z. Zheng, S.-Y. Zhou, J.-Y. Fang, O. Akdim, X.-Y. Ding, R. Oh, G.-S. Park, X. Huang, S.-G. Sun, Phase-dependent electrocatalytic nitrate reduction to ammonia on Janus Cu@Ni tandem catalyst. *ACS Catal.* **13**, 5098–5108 (2024).

20. J.-J. Zhang, Y.-Y. Lou, Z. Wu, X. J. Huang, S.-G. Sun, Spatially separated Cu/Ru on ordered mesoporous carbon for superior ammonia electrosynthesis from nitrate over a wide potential window. *J. Am. Chem. Soc.* **146**, 24966–24977 (2024).

21. Z. Cui, P. Zhao, H. Wang, C. Li, W. Peng, J. Liu, Tandem effect promotes MXene-supported dual-site Janus nanoparticles for high-efficiency nitrate reduction to ammonia and energy output through Zn-nitrate battery. *Adv. Mater.* **34**, 2410941 (2024).

22. K. D. Gilroy, H.-C. Peng, X. Yang, A. Ruditskiy, Y. Xia, Symmetry breaking during nanocrystal growth. *Chem. Commun.* **53**, 4530–4541 (2017).

23. J. Zeng, C. Zhu, J. Tao, M. Jin, H. Zhang, Z.-Y. Li, Y. Zhu, Y. Xia, Controlling the nucleation and growth of silver on palladium nanocubes by manipulating the reaction kinetics. *Angew. Chem. Int. Ed. Engl.* **51**, 2354–2358 (2012).

24. Q. N. Nguyen, C. Wang, Y. Shang, A. Janssen, Y. Xia, Colloidal synthesis of metal nanocrystals: From asymmetrical growth to symmetry breaking. *Chem. Rev.* **123**, 3693–3760 (2023).

25. H. Jia, Y. Yang, T. H. Chow, H. Zhang, X. Liu, J. Wang, C.-y. Zhang, Symmetry-broken Au–Cu heterostructures and their tandem catalysis process in electrochemical CO_2 reduction. *Adv. Funct. Mater.* **31**, 2101255 (2021).

26. S. Liu, Z. Sun, Q. Liu, L. Wu, Y. Huang, T. Yao, J. Zhang, T. Hu, M. Ge, F. Hu, Z. Xie, G. Pan, S. Wei, Unidirectional thermal diffusion in bimetallic Cu@Au nanoparticles. *ACS Nano* **8**, 1886–1892 (2014).

27. Y. Zheng, X. Zhong, Z. Li, Y. Xia, Successive, seed-mediated growth for the synthesis of single-crystal gold nanospheres with uniform diameters controlled in the range of 5–150 nm. *Part. Syst. Charact.* **31**, 266–273 (2014).

28. S.-H. Liu, K. A. Fichthorn, Interaction of alkylamines with Cu surfaces: A metal–organic many-body force field. *J. Phys. Chem. C* **121**, 22531–22541 (2017).

29. R. Zhang, M. Hummelgård, H. Olin, Simple synthesis of clay−gold nanocomposites with tunable color. *Langmuir* **26**, 5823–5828 (2010).

30. Q. Yang, H. Kong, L. Tang, Y. Ma, F. Liu, M. Liu, Y. Wang, P. Zhang, Y. Zheng, Au–Cu Janus nanostructures as NIR-II photothermal antibacterial agents. *ACS Appl. Nano Mater.* **7**, 20783–20792 (2024).

31. Z. Lyu, M. Xie, E. Aldama, M. Zhao, J. Qiu, S. Zhou, Y. Xia, Au@Cu core–shell nanocubes with controllable sizes in the range of 20–30 nm for applications in catalysis and plasmonics. *ACS Appl. Nano Mater.* **2**, 1533–1540 (2019).

32. Y. Shi, Z. Lyu, J. Liu, E. Chase, Y. Xia, Facile synthesis of Pd−Cu bimetallic twin nanocubes and a mechanistic understanding of the shape evolution. *ChemNanoMat* **6**, 386–391 (2020).

33. M. Homma, T. Sannomiya, Plasmonic Au–Cu alloy nanoparticles exhibiting order–disorder phase transitions for sensing and catalysis. *ACS Appl. Nano Mater.* **8**, 7662–7668 (2025).

34. Q. Gao, B. Yao, H. S. Pillai, W. Zang, X. Han, Y. Liu, S.-W. Yu, Z. Yan, B. Min, S. Zhang, H. Zhou, L. Ma, H. Xin, Q. He, H. Zhu, Synthesis of core/shell nanocrystals with ordered intermetallic single-atom alloy layers for nitrate electroreduction to ammonia. *Nat. Synth.* **2**, 624–634 (2023).

35. A. R. Denton, N. W. Ashcroft, Vegard's Law. *Phys. Rev. A* **43**, 3161–3164 (1991).

36. C. Wang, J. He, Y. Xia, Controlling the composition and elemental distribution of bi- and multi-metallic nanocrystals *via* dropwise addition. *Nat. Synth.* **3**, 1076–1082 (2024).

37. J. He, H. Yu, Y. Xia, Steady-state synthesis of colloidal metal nanocrystals. *Mater. Interfaces* **2**, 213–225 (2025).

38. S. G. Kwon, G. Krylova, P. J. Phillips, R. F. Kile, S. Chattopadhyay, T. Shibata, E. E. Bunel, Y. Liu, V. B. Prakapenka, B. Lee, E. V. Shevchenko, Heterogeneous nucleation and shape transformation of multicomponent metallic nanostructures. *Nat. Mater*. **14**, 215–223 (2015).

39. M. Tsuji, D. Yamaguchi, M. Matsunaga, M. J. Alam, Epitaxial growth of Au@Cu core−shell nanocrystals prepared using the PVP-assisted polyol reduction method. *Cryst. Growth Des.* **10**, 5129–5135 (2010).

40. K. K. Li, L. Kavalsky, M. Figueras-Valls, Y. Ding, M. Mavrikakis, Y. Xia, What makes Au nanospheres superior to octahedral and cubic counterparts for the deposition of a Pt monolayer shell? *J. Am. Chem. Soc.* **147**, 25273–25286 (2025).

41. R. Ferrando, J. Jellinek, R. L. Johnston, Nanoalloys: From theory to applications of alloy clusters and nanoparticles. *Chem. Rev*. **108**, 845–910 (2008).

42. S. Thota, Y. Wang, J. Zhao, Colloidal Au–Cu alloy nanoparticles: Synthesis, optical properties and applications. *Mater. Chem. Front.* **2**, 1074–1089 (2018).

43. H.-C. Peng, J. Park, L. Zhang, Y. Xia, Toward a quantitative understanding of symmetry reduction involved in the seed-mediated growth of Pd nanocrystals. *J. Am. Chem. Soc.* **137**, 6643–6652 (2015).

44. E. Y. El koraychy, R. Ferrando, Growth pathways of Cu shells on Au and AuCu seeds: interdiffusion, shape transformations, strained shells and patchy surfaces. *Nanoscale Adv.* **5**, 5838–5849 (2023).

45. P. Mulvaney, Surface plasmon spectroscopy of nanosized metal particles. *Langmuir* **12**, 788–800 (1996).

46. Y. Xin, K. Yu, L. Zhang, Y. Yang, H. Yuan, H. Li, L. Wang, J. Zeng, Copper-based plasmonic catalysis: recent advances and future perspectives. *Adv. Mater.* **33**, e2008145 (2021).

47. S. Kim, J.-M. Kim, J.-E. Park, J.-M. Nam, Nonnoble-metal-based plasmonic nanomaterials: recent advances and future perspectives. *Adv. Mater.* **30**, e1704528 (2018).

48. S.-H. Wu, D.-H. Chen, Synthesis of high-concentration Cu nanoparticles in aqueous CTAB solutions. *J. Colloid Interface Sci.* **273**, 165–169 (2004).

49. K. K. Li, C.-Y. Wu, T.-H. Yang, D. Qin, Y. Xia, Quantification, exchange, and removal of surface ligands on noble-metal nanocrystals. *Acc. Chem. Res.* **56**, 1517–1527 (2023).

50. N. Kruse, S. Chenakin, XPS characterization of Au/TiO_2 catalysts: binding energy assessment and irradiation effects. *Appl. Catal. Gen.* **391**, 367–376 (2011).

51. Z. Chen, T. Balankura, K. A. Fichthorn, R. M. Rioux, Revisiting the polyol synthesis of silver nanostructures:Role of chloride in nanocube formation. *ACS Nano* **13**, 1849–1860 (2019).

52. S. Xie, H.-C. Peng, N. Lu, J. Wang, M. J. Kim, Z. Xie, Y. Xia, Confining the nucleation and overgrowth of Rh to the {111} facets of Pd nanocrystal seeds: The roles of capping agent and surface diffusion. *J. Am. Chem. Soc.* **135**, 16658–16667 (2013).

53. J.-Y. Fang, Q.-Z. Zheng, Y.-Y. Lou, K.-M. Zhao, S.-N. Hu, G. Li, O. Akdim, X.-Y. Huang, S.-G. Sun, Ampere-level current density ammonia electrochemical synthesis using CuCo nanosheets simulating nitrite reductase bifunctional nature. *Nat. Commun.* **13**, 7899 (2022).

54. H. Liu, Y. Jia, X. Huang, Y. Liu, Q. Yang, Z. Chen, J. Xu, AuCu nanodendrite for enhancing electrocatalytic nitrate reduction applications *via* two-stage microfluidic fabrication strategy. *ACS Catal.* **15**, 1230–1241 (2025).

55. Y. Xiao, X. Tan, B. Du, Y. Guo, W. He, H. Cui, C. Wang, Strained Au skin on mesoporous intermetallic $AuCu_3$ nanocoral for electrocatalytic conversion of nitrate to ammonia across a wide concentration range. *Angew. Chem. Int. Ed. Engl.* **136**, e202408758 (2024).

56. L. Sun, H. Lv, J. Xiao, B. Liu, Enzymatic mesoporous metal nanocavities for concurrent electrocatalysis of nitrate to ammonia coupled with polyethylene terephthalate upcycling. *Adv. Mater.* **36**, e2402767 (2024).

57. L. Sun, H. Yao, F. Jia, Y. Wang, B. Liu, Intermediate confinement for selective ammonia electrosynthesis from nitrate on robust mesoporous metal catalysts. *Adv. Energy Mater.* **13**, 2302274 (2023).

58. J. Hutter, M. Iannuzzi, F. Schiffmann, J. VandeVondele, CP2K: Atomistic simulations of condensed matter systems. *WIREs Comput. Mol. Sci*. **4**, 15–25 (2014).

59. J. P. Perdew, A. Ruzsinszky, G. I. Csonka, O. A. Vydrov, G. E. Scuseria, L. A. Constantin, X. Zhou, K. Burke, Restoring the density-gradient expansion for exchange in solids and surfaces. *Phys. Rev. Lett.* **100**, 136406 (2008).

60. J. VandeVondele, J. Hutter, Gaussian basis sets for accurate calculations on molecular systems in gas and condensed phases. *J. Chem. Phys.* **127**, 114105 (2007).

61. S. Grimme, J. Antony, S. Ehrlich, H. Krieg, A consistent and accurate ab *initio* parametrization of density functional dispersion correction (DFT-D) for the 94 elements H-Pu. *J. Chem. Phys.* **132**, 154104 (2010).

62. J. P. Perdew, K. Burke, M. Ernzerhof, Generalized gradient approximation made simple. *Phys. Rev. Lett.* **77**, 3865–3868 (1996).

63. Q. Gao, H. S. Pillai, Y. Huang, S. Liu, Q. Mu, X. Han, Z. Yan, H. Zhou, Q. He, H. Xin, H. Zhu, Breaking adsorption-energy scaling limitations of electrocatalytic nitrate reduction on intermetallic CuPd nanocubes by machine-learned insights. *Nat. Commun.* **13**, 2338 (2022).

64. M. Verstraete, X. Gonze, Smearing scheme for finite-temperature electronic-structure calculations. *Phys. Rev. B* **65**, 035111 (2001).

65. K. Momma, F. Izumi, *VESTA 3* for three-dimensional visualization of crystal, volumetric and morphology data. *J. Appl. Cryst.* **44**, 1272–1276 (2011).

66. M. Yu, D. R. Trinkle, Accurate and efficient algorithm for Bader charge integration. *J. Chem. Phys.* **134**, 064111 (2011).

67. A. A. Peterson, F. Abild-Pedersen, F. Studt, J. Rossmeisl, J. K. Nørskov, How copper catalyzes the electroreduction of carbon dioxide into hydrocarbon fuels. *Energ. Environ. Sci.* **3**, 1311–1315 (2010).

68. T. Lu, Q. Chen, Shermo: A general code for calculating molecular thermochemistry properties. *Comput. Theor. Chem.* **1200**, 113249 (2021).

69. C. P. Kelly, C. J. Cramer, D. G. Truhlar. Aqueous solvation free energies of ions and ion−water clusters based on an accurate value for the absolute aqueous solvation free energy of the proton. *J. Phys. Chem. B* **110**, 16066–16081 (2006).

70. Y. Zhao, N. E. Schultz, D. G. Truhlar. Design of density functionals by combining the method of constraint satisfaction with parametrization for thermochemistry, thermochemical kinetics, and noncovalent interactions. *J. Chem. Theory Comput.* **2**, 364–382 (2006).

71. V. A. Rassolov, M. A. Ratner, J. A. Pople, P. C. Redfern, L. A. Curtiss, 6-31G* basis set for third-row atoms. *J. Comput. Chem.* **22**, 976–984 (2001).

72. F. Neese, Software update: The ORCA program system—Version 5.0. *WIREs Comput. Mol. Sci.* **12**, e1606 (2022).

73. A. V. Marenich, C. J. Cramer, D. G. Truhlar. Universal solvation model based on solute electron density and on a continuum model of the solvent defined by the bulk dielectric constant and atomic surface tensions. *J. Phys. Chem. B* **113**, 6378–6396 (2009).

74. Y. Wang, W. Zhou, R. Jia, Y. Yu, B. Zhang, Unveiling the activity origin of a copper-based electrocatalyst for selective nitrate reduction to ammonia. *Angew. Chem. Int. Ed. Engl.* **59**, 5350–5354 (2020).

75. Q. Hu, Y. Qin, X. Wang, Z. Wang, X. Huang, H. Zheng, K. Gao, H. Yang, P. Zhang, M. Shao, C. He, Reaction intermediate-mediated electrocatalyst synthesis favors specified facet and defect exposure for efficient nitrate-ammonia conversion. *Energ. Environ. Sci.* **14**, 4989–4997 (2021).

76. W. Gao, K. Xie, J. Xie, X. Wang, H. Zhang, S. Chen, H. Wang, Z. Li, C. Li, Alloying of Cu with Ru enabling the relay catalysis for reduction of nitrate to ammonia. *Adv. Mater.* **35**, e2202952 (2023).

77. J. Wang, Y. Wang, C. Cai, Y. Liu, D. Wu, M. Wang, M. Li, X. Wei, M. Shao, M. Gu, Cu-doped Iron oxide for the efficient electrocatalytic nitrate reduction reaction. *Nano Lett.* **23**, 1897–1903 (2023).

78. L. Sun, X. Min, J. Wang, Y. Yang, S. Han, D. Fan, Y. Jing, D. Xu, W. Wang, B. Liu, Enabling high performance in a positive potential of nitrate-to-ammonia electrocatalysis over mesoporous core@shell Cu_2O/Cu@PdCu nanozyme. *Adv. Mater.* **37**, e2503291 (2025).
